# Copper Pairing in the Mordenite Framework as a Function of the Cu^I^/Cu^II^ Speciation

**DOI:** 10.1002/anie.202109705

**Published:** 2021-11-03

**Authors:** Gabriele Deplano, Andrea Martini, Matteo Signorile, Elisa Borfecchia, Valentina Crocellà, Stian Svelle, Silvia Bordiga

**Affiliations:** ^1^ Department of Chemistry NIS and INSTM Reference Centre Università di Torino Via G. Quarello 15 I-10135 and Via P. Giuria 7, I-10125 Torino Italy; ^2^ Smart Materials Research Institute Southern Federal University Sladkova Street 174/28 344090 Rostov-on-Don Russia; ^3^ Department of Chemistry SMN Centre for Materials Science and Nanotechnology University of Oslo N-0315 Oslo Norway

**Keywords:** copper, EXAFS, methane-to-methanol conversion, mordenite, wavelet transform, XANES

## Abstract

A series of gas‐phase reactants is used to treat a Cu‐exchanged mordenite zeolite with the aim of studying the influence of the reaction environment on the formation of Cu pairs. The rearrangement of Cu ions to form multimeric sites as a function of their oxidation state was probed by X‐ray absorption spectroscopy (XAS) and also by applying advanced analysis through wavelet transform, a method able to specifically locate Cu–Cu interactions also in the presence of overlapping contributions from other scattering paths. The nature of the Cu‐oxo species formed upon oxidation was further crosschecked by DFT‐assisted fitting of the EXAFS data and by resonant Raman spectroscopy. Altogether, the Cu^I^/Cu^II^ speciation clearly correlates with Cu proximity, with metal ion pairs quantitatively forming under an oxidative environment.

## Graphical abstract

As evidenced by advanced X‐ray absorption spectroscopy analysis, Cu‐pairing in copper‐exchanged mordenite is favored by the +2 oxidation state. The optimal Cu–Cu distance in the oxidized state favors the formation of dimeric mono‐μ‐oxo dicopper sites.

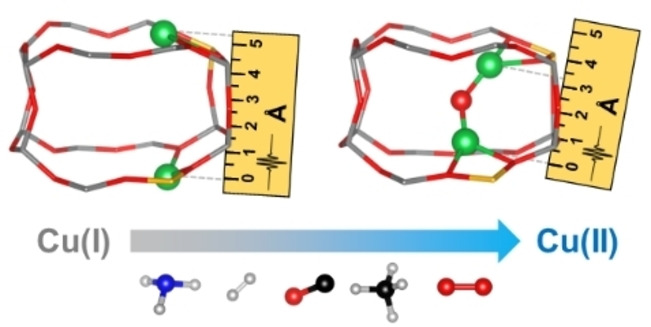

## Introduction

Within the paradigm of an improved use of fossil resources, strategies facilitating the exploitation of many small/remote natural gas sources (including biogas) is of utmost importance.[^1^, ^2^] In particular, chemical processes able to transform methane (the main constituent of natural gas) into liquid analogues are desirable for a simplified handling and transportation. The presently implemented syngas‐based technologies are energy intensive processes, thus economically viable only for large‐scale applications.^[3]^ As an alternative, the direct conversion of methane to methanol (DMTM) via partial oxidation represents a promising route. In analogy to biological systems (e.g. methanotrophic bacteria hosting the pMMO enzymes),[^4^, ^5^, ^6^] several Cu‐based catalysts for DMTM have been developed and studied.^[7]^ Among them, Cu‐exchanged zeolites have received much attention after the discovery of their activity in DMTM by Schoonheydt's group in 2005.^[8]^ Many different topologies and compositions (in terms of Si/Al and Cu/Al ratios) have been explored, aiming at finding structure–activity relationships.^[9]^ A combination of experimental and theoretical techniques has been applied to identify the active species responsible for this reactivity, and many mechanisms have been proposed to explain their formation and outstanding selectivity. Moreover, different reaction conditions heavily impact the speciation of Cu inside the zeolite framework, thus influencing DMTM.^[10]^ Overall, the Cu^I^/Cu^II^ redox cycle and the local coordination environment around Cu sites are the key features for understanding these complex systems.

In this context, opportune gas‐phase reactants (e.g. NH_3_) affect both Cu speciation and framework distribution when used prior to oxidation, as the coordinative nature of NH_3_ enhances the Cu cations’ mobility.^[11]^ Accordingly, the Cu speciation in the framework changes,^[12]^ as well as the average Cu–Cu distance, in turn conditioning the formation of active species. Indeed, these are supposedly multinuclear Cu sites bridged by O atoms; different Cu–Cu distances could affect oxidation pathways and drive towards different speciation. Thereby, understanding the effect of the redox history of a Cu‐zeolite on the final speciation of Cu sites (including Cu‐oxo species) is relevant for optimizing both DMTM catalysts and reaction protocols. Accordingly, a selection of reducing agents has been used to activate a Cu‐MOR sample subsequently characterized through XAS, aiming at probing the oxidation state and local environment of the resulting species. Furthermore, the application of Wavelet Transform (WT) Analysis^[13]^ on these data allowed us correlating the existence of Cu–Cu interactions with the fraction of reduced/oxidized Cu species. Through DFT‐supported fitting of the EXAFS data, specific Cu‐oxo species were identified.

## Results and Discussion

**Figure 1:**
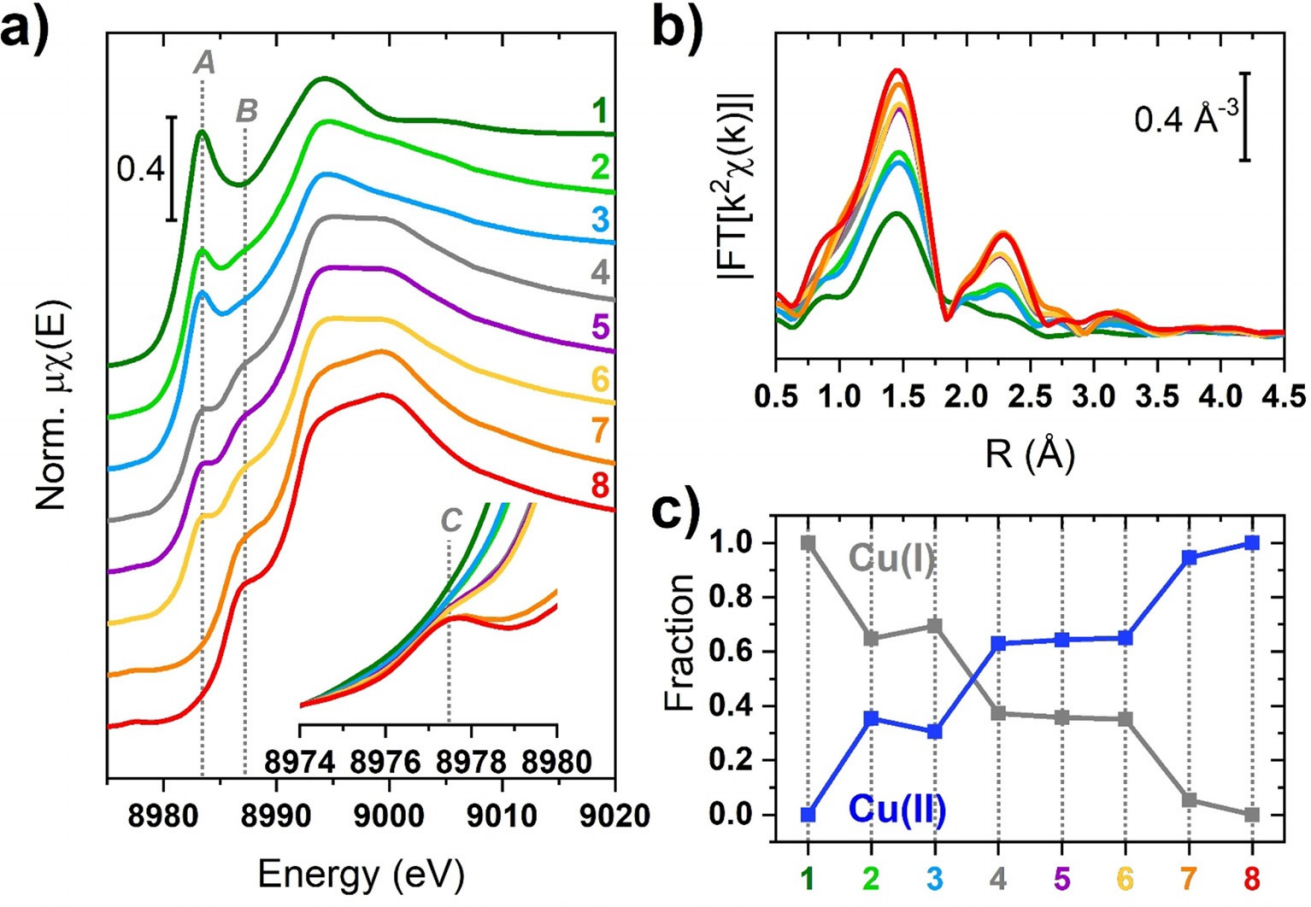
a) XANES spectra and b) k^2^‐weighted module of FT‐EXAFS for Cu‐MOR; c) linear combination fit of XANES spectra (samples 1 and 8 taken as references): 1) reduced in NH_3_ at 500 °C; 2) reduced in NH_3_ at 250 °C; 3) reduced in H_2_ at 250 °C; 4) reduced in CO at 250 °C; 5) reduced in CH_4_ at 250 °C; 6) self‐reduced by vacuum dehydration at 500 °C; 7) directly oxidized in O_2_ at 500 °C; and 8) pre‐reduced in NH_3_ and oxidized in O_2_ at 500 °C. In the inset of (a), the magnification of the weak Cu^II^ 1s→3d transition is reported. Vertical dotted lines highlight the Cu^I^ 1s→4p (A); Cu^II^ 1s→4p (B); and Cu^II^ 1s→3d (C) transitions. The errors affecting the Cu^I^/Cu^II^ fractions obtained from LCF are as low as 0.01.

In this work, we focus on an ad hoc synthesized Cu‐MOR (Si/Al=8.22 and Cu/Al=0.27, atomic ratios from EDX), prepared and characterized as described in Section S1 of the Supporting Information (SI). Basic characterization data (powder XRD and N_2_ adsorption isotherm at 77 K) are provided in Figure S1 of the SI. We relied on a home‐made material rather than on a commercial one, since Cu‐MOR samples we previously studied^[14]^ have been proved to contain traces of TiO_2_ (anatase polymorph), as shown in Figure S2 of the SI, making it unsuitable for Raman and optical characterization. Figure 1 a and c shows the ex situ XAS data collected at RT for the Cu‐MOR treated according to Section S1.1. Each sample was subjected to a specific gas‐phase redox treatment. Reduction in NH_3_, H_2_, CO and CH_4_ was performed at 250 °C. A sample deeply reduced in NH_3_ at 500 °C was also prepared. Regardless the reduction treatment, each sample was subsequently outgassed at 500 °C to ensure complete desorption of species possibly coordinating the Cu ions. Oxidized samples were produced by exposing the sample, possibly pre‐reduced in NH_3_ at 500 °C, to pure O_2_ at 500 °C. Finally, a sample that just underwent vacuum dehydration (thus triggering self‐reduction) was considered. Given the edge energy position and the observed XANES features, any contributions from metallic Cu, even in the form of small Cu^0^ clusters, can be safely ruled out over the whole set of investigated samples (see also Figure S3). The XANES spectrum in Figure 1 a, belonging to the sample reduced at 500 °C in the presence of NH_3_, shows no traces of the pre‐edge 1s*→*3d transition arising at ca. 8978 eV (see the inset in Figure 1 a), typical of Cu^II^ ions. Conversely, it is characterized by a prominent 1s→4p rising‐edge peak located at ca. 8983 eV and by a low intensity in the white line (WL) region, typical of a site with a low coordination number. In the limit of the energy resolution, these spectral features infer the existence of a quasi‐linear pure Cu^I^ site, in accordance with previous studies, also involving model compounds.[^2^, ^10^, ^14^, ^15^, ^16^, ^17^, ^18^, ^19^] This interpretation is in line with the phase‐uncorrected FT‐EXAFS spectrum (Figure 1 b): the first‐shell maximum is observed at 1.5 Å, with a shoulder extending within 1.8–2.6 Å. The low intensity of the first‐shell peak agrees with a twofold coordinated Cu^I^ center, most likely with framework oxygen atoms. The XANES spectra of the samples treated in NH_3_ and in H_2_ at 250 °C are still dominated by a Cu^I^ species, but the presence a minor fraction of Cu^II^ sites is indicated by the appearance of the Cu^II^ 1s→4p transition at ca. 8987 eV. Subtle modifications are also noted in the pre‐edge range, pointing to a trace of the Cu^II^ 1s→3d peak. Yet, within the available energy resolution, we cannot conclusively comment about this inherently weak spectral feature in samples 2 and 3. Consistently, if compared to the NH_3_ 500 °C state, the EXAFS spectrum exhibits a more intense first‐shell peak and a structured second‐shell peak, consistent with the presence of Cu^II^ sites interacting with the framework and having a higher coordination number. The XANES spectra corresponding to the samples reduced in CO, CH_4_ and self‐reduced (SR) are almost mutually identical.

From these profiles an increase in the relative contribution from Cu^II^ centers is clear, determining the abatement of the Cu^I^ 1s→4p transition and causing the increase of the Cu^II^ 1s→3d and 1s→4p ones. Overall, XANES indicates an almost equal Cu^I^/Cu^II^ fraction with a potential slight preference for Cu^II^ in these samples. The related EXAFS spectra show the same first‐ and second‐shell features described before, but more intense due to the larger fraction of Cu^II^ sites.

Finally, in agreement with the literature,[^10^, ^14^, ^19^, ^20^, ^21^, ^22^, ^23^] the XANES spectra of the samples oxidized at 500 °C account for a largely dominant Cu^II^ oxidation state. The Cu^I^ 1s→4p transition is absent in the XANES of both oxidized samples, inferring a pure Cu^II^ state. The corresponding EXAFS spectra are consistent with three/fourfold O‐ligated Cu^II^, located at well‐defined ion‐exchange sites in the zeolite framework.[^10^, ^12^] The second shell appears well‐structured and it stems mainly from the Cu–Al/Si and Cu–Cu single scattering (SS) contributions.

**Figure 2:**
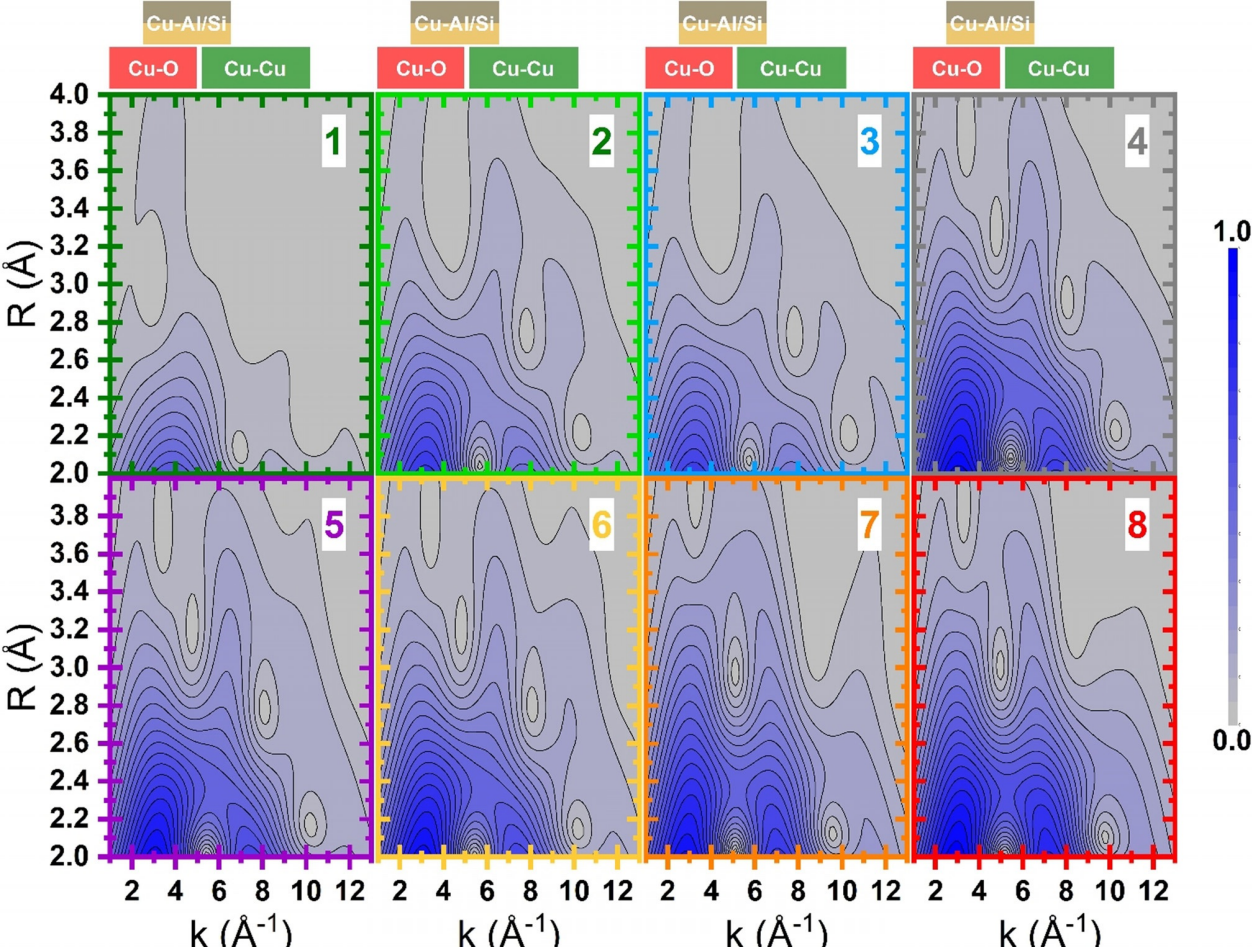
Wavelet transform representation of the EXAFS signal for Cu‐MOR: 1) reduced in NH_3_ at 500 °C; 2) reduced in NH_3_ at 250 °C; 3) reduced in H_2_ at 250 °C; 4) reduced in CO at 250 °C; 5) reduced in CH_4_ at 250 °C; 6) self‐reduced by vacuum dehydration at 500 °C; 7) directly oxidized in O_2_ at 500 °C; and 8) pre‐reduced in NH_3_ and oxidized in O_2_ at 500 °C. Boxes in the upper part of the figure highlight the region of maximum intensity in k for Cu–O, Cu–Si/Al and Cu–Cu scattering paths. Vertical lines highlight the position of maximum in k for Cu–Cu scattering path.

**Figure 3:**
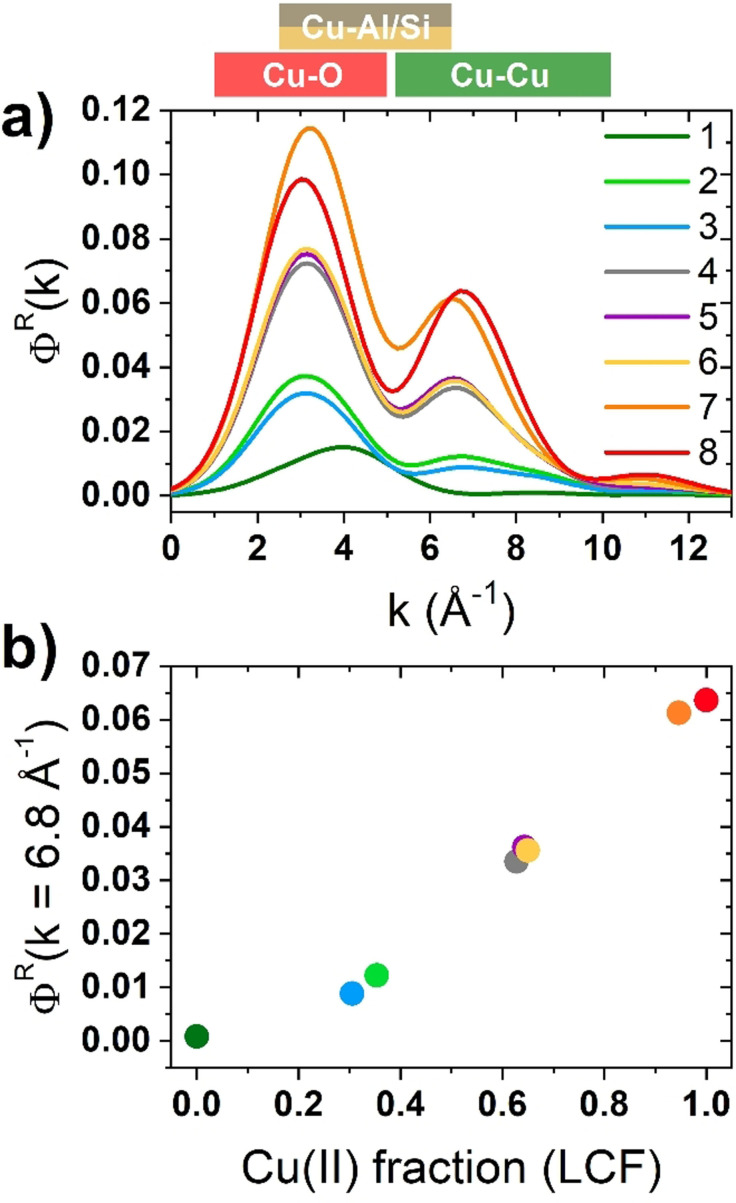
a) Power density function *Φ* ^*R*^ of each WT representation, integrated over the 2.0–4.0 Å range, for Cu‐MOR: 1) reduced in NH_3_ at 500 °C; 2) reduced in NH_3_ at 250 °C; 3) reduced in H_2_ at 250 °C; 4) reduced in CO at 250 °C; 5) reduced in CH_4_ at 250 °C; 6) self‐reduced by vacuum dehydration at 500 °C; 7) directly oxidized in O_2_ at 500 °C; and 8) pre‐reduced in NH_3_ and oxidized in O_2_ at 500 °C. b) Value of *Φ* ^*R*^ at *k*=6.8 Å^−1^ (highlighted by the vertical dotted line in (a) as a function of the Cu^II^ fraction as obtained from LCF of XANES spectra (see Figure 1 c). Boxes in the upper part of the Figure highlight the region of maximum intensity in k for Cu–O, Cu–Si/Al and Cu–Cu scattering paths. Vertical lines highlight the position of maximum in k for Cu–Cu scattering path.

**Figure 4:**
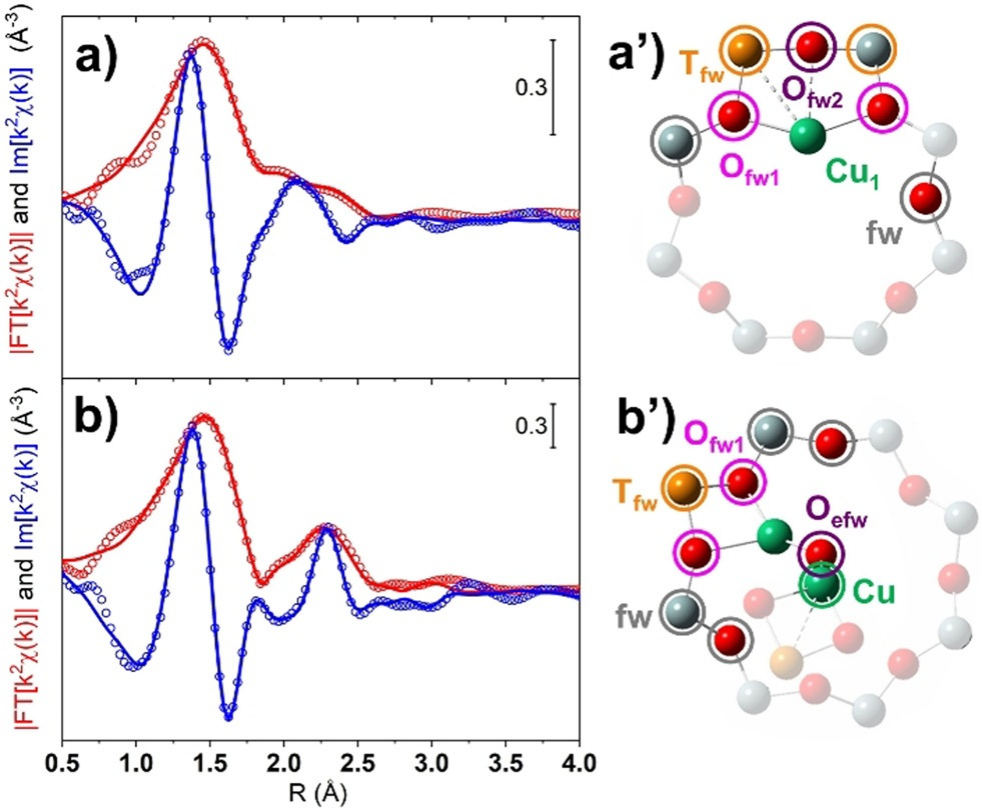
Phase‐uncorrected (red) modulus and imaginary part (blue) of the experimental and best‐fit FT EXAFS spectra for a) the most reduced sample (i.e. treated at 500 °C in NH_3_, sample 1 in Figure 1); and b) further oxidized in pure O_2_ at 500 °C (sample 8 in Figure 1). Experimental data are shown as open circles, the best fits as solid lines. The structural models adopted in the fit are shown in (a′) (2[Cu^I^]^+^, only one Cu ion of the pair showed here for clarity) and (b′) (([Cu^II^‐O‐Cu^II^]^2+^). Atoms color code: Cu, green; Si, gray; Al, yellow; O, red. The atoms included in the fitting model are shown in bright colors. The entire structure of the models is reported in Figure S9a,b.

The amount of Cu^I^ and Cu^II^ species in each XANES spectrum was obtained by linear combination fit (LCF)^[24]^ on the normalized XANES (Figure 1 a), in the 8975–9020 eV range. The number of chemical species was defined through a Principal Component Analysis[^25^, ^26^, ^27^] (see Figure S4 of the SI) and set to two. The XANES spectra of samples reduced in NH_3_ (sample 1) and pre‐reduced in NH_3_ and then oxidized in O_2_ at 500 °C (sample 8) were chosen as standards for the least‐square procedure.^[28]^ The XANES LCF provided a very small *R*_factor_ (0.04 %) concerning the reconstruction of the experimental XANES, indicating that the selected references are suitable to reproduce each single XANES spectrum of the dataset; a direct comparison between experimental and LCF curves can be found in Figure S5. The Cu^I^–Cu^II^ fraction, retrieved by this approach, is reported in Figure 1 c. The samples treated with NH_3_ and H_2_ at 250 °C show an almost equivalent larger abundance (about 65 %) of Cu^I^ sites. Instead, the self‐reduced sample and the ones treated with CO or CH_4_ show a lower fraction of Cu^I^ sites (below 40 %), becoming closely nil in the sample directly oxidized at 500 °C (<5 %). Due to its sensitivity to the chemical nature of the scatterers surrounding the absorbing atom, we employed the WT approach to provide a more robust description of high *R* EXAFS features.[^13^, ^29^] All the WT representations show a main lobe at low *R* values (*Δk*: 0.0–12.5 Å^−1^ and Δ*R*: 0.5–2.0 Å), as observed on the full two dimensional *(k*,*R)* maps in Figure S6. As previously described, this feature is due to the SS contributions arising from the (extra‐)framework oxygen atoms located in the first coordination shell of the Cu centers. Figure 2 shows, for each treatment, the region of the WT map involving the EXAFS second‐ and third‐shells (Δ*k*: 0.0–12.5 Å^−1^ and Δ*R*: 2.0–4.0 Å), where Cu–Cu contributions are expected. The WT map of the sample reduced in NH_3_ at 500 °C only shows a weak lobe at *k* values around 4.0 Å^−1^. In this region, the backscattering amplitude factor terms for Cu–O and Cu–Al/Si SS have their maxima, largely overlapped (see Figure S7). The WT map confirms that the shoulder appearing in the FT‐EXAFS for this sample derives from weak scattering paths involving the farther framework O and Si/Al atoms of the low coordinated Cu^I^ site. In the WT maps of samples reduced at 250 °C in NH_3_ and H_2_, the low k‐region becomes more structured. A second lobe appears at higher k values (Δ*k*: 5.0–7.0 Å^−1^ and Δ*R*: 2.0–4.0 Å), arising from intensified contributions of scattering paths among Si/Al and Cu^II^ sites, superimposed to contributions from the lattice O farther from Cu centers. The presence of a small Cu–Cu contribution cannot be excluded, albeit overshadowed by signal from the framework atoms. In fact, a weak ridge is observed at 7.0 Å^−1^, where the backscattering amplitude factor for a Cu–Cu SS has its maximum. The latter presents increased intensity in the WT maps of samples reduced in CO, CH_4_ and SR. Finally, this feature reaches its maximum intensity for both samples oxidized in O_2_ at 500 °C, that is, when the content of Cu^II^ sites is the highest.[^13^, ^30^] At its maximum development, this sub‐lobe extends over a rather broad R‐space range, pointing to a relatively high level of structural disorder in the Cu–Cu interatomic distance distribution. In order to comparatively assess the presence of Cu–Cu scattering contributions through the investigated states, similarly to some previous reports,[^13^, ^30^, ^31^] we computed the power density function *Φ* ^*R*^(*k*) of each WT representation. This quantity was obtained integrating the squared modulus of the WT over the *R* range within 2.0 and 4.0 Å, which ensures the inclusion, if present, of whatever Cu–Cu path contribution. Figure 3 a shows the results of these calculations. Herein, a first peak is located for all the states between 0.0–5.5 Å^−1^, ascribed to the WT low‐k sub‐lobes, which collectively account for the contribution due to O, Si and Al atomic neighbors surrounding the Cu centers. The second, weaker peak (6.0–8.0 Å^−1^) is ascribable to Cu–Cu contributions, reflecting the high‐k sub‐lobe in the WT maps discussed before. The intensity of this peak, as measured at its maximum at 6.8 Å^−1^ for each of the considered states, nicely correlates with the Cu^II^ fraction as previously determined by XANES LCF. Such a result, depicted in Figure 3 b, quantitatively confirms the preference of Cu^II^ toward the formation of multimeric species, whereas Cu^I^ ions remain preferentially separated. The approach of Cu ions in their oxidized form, coherently observed in an oxidative environment, is most probably accompanied by the formation of Cu‐oxo species, supposedly active in DMTM. Thus, their exact identification is of utmost interest toward a better understanding of the methane oxidation process (despite not explicitly investigated here). Furthermore, the observation of fingerprints of specific Cu‐oxo species also supports the qualitative analysis of Cu–Cu distances as inferred by EXAFS data obtained via Fourier or Wavelet transform. Thereby, an EXAFS fitting procedure was carried out. In particular, we focused our analysis on the two extreme cases: i) at the most reduced sample, that is, treated at 500 °C in NH_3_ (sample 1 in Figure 1); and ii) further oxidized in pure O_2_ at 500 °C (sample 8 in Figure 1). As structural guess we created, on the basis of the recent literature,[^10^, ^13^, ^14^, ^17^, ^22^, ^32^] four DFT models as described in detail in the Section S5 of SI. The Cu(‐oxo) models were hosted in the eight‐membered rings of the side pocket of MOR structure, where the siting of Al atoms (i.e. the anchoring site for the Cu ions) was selected on the basis of a systematic prescreening of all possible configurations (see Figure S8 for the considered Al substitutional sites and Table S1 for main outcomes). Four Cu(‐oxo) structures were considered (graphically represented in Figure S9): a couple of Cu^I^ ions (labeled 2[Cu^I^]^+^), a mono‐μ‐oxo dicopper site ([Cu^II^‐O‐Cu^II^]^2+^) and two peroxo dicopper sites, owning a different spatial configuration ([Cu^II^‐OO‐Cu^II^]^2+^_sideon_ and [Cu^II^‐OO‐Cu^II^]^2+^_endon_). The fit on the reduced sample was performed considering the 2[Cu^I^]^+^, whereas that for the oxidized state was attempted with all the aforementioned Cu‐oxo models. Nonetheless, only the fit based on the [Cu^II^‐O‐Cu^II^]^2+^ structure was sufficiently in agreement with the experimental data (see Section S6 of the SI) and will be discussed herein. The EXAFS fit results, summarized in Figure 4 and Table S2 of the SI (fixed coordination numbers for the 2[Cu^I^]^+^ and the [Cu^II^‐O‐Cu^II^]^2+^ DFT models are given in Table S3 and S4, respectively) and the in Table, properly reproduce the experimental spectra (*R*_factor_ lower than 1 % in both the cases) and provided a set of physically reliable optimized parameters. Results obtained for a fit attempt performed with the [Cu^II^‐OO‐Cu^II^]^2+^_sideon_ structural model are reported in Table S5 (fixed coordination numbers in Table S6), though some physically inconsistent parameters were obtained. The fitting strategies and parametrization adopted are described in Section S6.1 of the SI, while the individual EXAFS path contributions are shown in Figure S10 and S11. Raw data and best‐fit in k‐space are shown in Figure S12.

Focusing on the sample reduced in NH_3_ at 500 °C, the EXAFS first‐shell feature mostly originates from the SS paths involving the framework oxygen atoms sited close to the Cu absorbers. The latter were found to be approximately in phase (see Figure S10) with an average Cu–O_fw1_ distance refined at 1.93±0.01 Å. The broadening of the first‐shell peak toward longer distances can be ascribed to a second type of O framework atoms at a slightly longer distance from the absorber (i.e. Cu–O_fw2_). The contribution from farther framework atoms (O, Al and Si) is instead weak since, due to the high heterogeneity of the Cu‐sites, these paths are in antiphase to each other, causing the abatement of their EXAFS signals (see Figure S10). Indeed, high R‐values features are absent, in accordance with the related WT representation (see Figure 2, panel 1). Finally, the interaction involving the Cu–Cu path was not included in the fit, since the interatomic distance among the two Cu^I^ atoms in the 2[Cu^I^]^+^ DFT model is >5.0 Å, that is, outside the limit of detectability of FT‐EXAFS analysis for this case. Considering the EXAFS spectrum of Cu‐MOR activated in O_2_ at 500 °C after pre‐reduction in NH_3_ at 500 °C, the first‐shell of the experimental FT‐profile is successfully reproduced considering two subs‐hell of O neighbors in the first‐coordination sphere, involving framework (O_fw1_) and extraframework (O_efw_, that is, involved in the formation of Cu‐oxo species) O atoms. These two families of O atoms contribute in partial antiphase (see Figure S11) at 1.93±0.02 and 1.97±0.02 Å, respectively. The second maximum of the FT‐EXAFS is effectively modelled by the SS contribution of a single T_fw_ (Al) atom at a distance of 2.68±0.01 Å from the Cu absorber. In the high‐R range, the contribution from farther framework O and Si atoms (fw) is observed too. Finally, a Cu–Cu contribution is refined at 3.28±0.08 Å (ca. 2.88 Å in the phase uncorrected FT‐EXAFS plot), consistent with previous WT‐EXAFS fitting results on oxidized Cu‐MOR,^[13]^ as well as conventional EXAFS fitting on Cu‐CHA.[^10^, ^30^] Based on a more symmetrical mono‐μ‐oxo dicopper site model, Sushkevich et al.^[29]^ reported instead shorter Cu–Cu separations around 2.85 Å, yet associated with minority Cu‐species, as indicated by coordination numbers of ca. 0.3. Not surprisingly, our EXAFS analysis revealed a relatively high Debye–Waller factor associated with Cu–Cu scattering path, properly reflecting the broad intensity distribution observed for the Cu–Cu sub‐lobe in the WT maps (Figure 2). The small deviation (ca. −0.02 Å) of the fitted *R_Cu_* from the DFT distance fully supports the choice of this structure for the EXAFS fitting refinement, in line with the occurrence of such type of [Cu^II^‐O‐Cu^II^]^2+^cores as a dominant, although not exclusive, configuration under the adopted pretreatment conditions.

## Conclusion

In summary, a sample of Cu‐mordenite was systematically treated with a broad set of gas‐phase reactants to gain specific information on the influence of different redox‐active molecules on Cu pairing. XAS was employed to simultaneously probe the oxidation state and the proximity of the Cu sites as a function of different redox treatments. In addition, the WT approach (augmented by the power density function analysis) was demonstrated being an irreplaceable tool toward the selective assessment of Cu–Cu contributions in the XAS dataset, showing a relation between the oxidation state of the metal center and the proximity of Cu sites. For the first time, the presence of Cu pairs was quantitatively correlated to the fraction of oxidized Cu^II^ sites present in the sample. DFT‐assisted EXAFS fitting further allowed identifying the fingerprints of the Cu‐oxo species formed after oxidative treatment as due to dimeric Cu‐O‐Cu sites (also confirmed by resonant Raman spectroscopy, see Figure S13). As multiple Cu sites have been proved to work cooperatively through the redox pathways that lead both to activation of the material and reaction with methane, reliably monitoring the specific interactions between the metal sites is essential to understand the mechanism underlying these processes. In addition, exploring how different reactants interact with these materials help in identifying relationships between the activation protocol and the reaction performances. Exploring in a rational way the range of variability that characterizes these systems (topology, composition, reactants, temperatures) with tools capable to detect such specific features and trends will be the key to a better understanding of selective DMTM process, pointing toward ad hoc engineering of catalysts and reaction protocols that could maximize selectivity and productivity.

## Conflict of interest

The authors declare no conflict of interest.

## Supporting information

As a service to our authors and readers, this journal provides supporting information supplied by the authors. Such materials are peer reviewed and may be re‐organized for online delivery, but are not copy‐edited or typeset. Technical support issues arising from supporting information (other than missing files) should be addressed to the authors.

Supporting Information
