# Supplementary material for: Copper Pairing in the Mordenite Framework as a Function of the CuI/CuII Speciation
Source: Angew Chem Int Ed Engl. 2021 Nov 3;60(49):25891–6. doi: 10.1002/anie.202109705 (PMC9298398; doi:10.1002/anie.202109705)
Supplement: Supplementary file 1 — Supporting Information [file ANIE-60-25891-s001.pdf]

Angewandte Chemie
Eine Zeitschrift der Gesellschaft Deutscher Chemiker

Supporting Information

Copper Pairing in the Mordenite Framework as a Function of the Cu^{I}/Cu^{II} Speciation

Gabriele Deplano+, Andrea Martini+, Matteo Signorile, Elisa Borfecchia, Valentina Crocellà, Stian Svelle, and Silvia Bordiga*

anie_202109705_sm_miscellaneous_information.pdf

Table of Contents

S1. Experimental details	S1
S1.1. Materials	S1
S1.2. Characterization	S3
S2. Characterization of the home-made Cu-MOR samples	S5
S3. Details on XANES analysis	S7
S3.2 Comparison of XANES spectra for selected Cu-MOR and Cu metal foil	S7
S3.2 PCA analysis of the XANES dataset	S8
S3.2 Comparison between experimental and LCF XANES spectra	S10
S4. EXAFS Wavelet Transform Analysis	S11
S4.1. Full range wavelet transform representation	S11
S4.2. Representative FEFF-derived Backscattering amplitude Factors	S12
S5. Computational screening on Cu pairs distribution in 8-MR side pockets	S13
S6. EXAFS analysis	S18
S6.1 Fitting strategy	S18
S6.2 EXAFS best fits	S20
S6.3 Experimental and best-fit EXAFS spectra in k-space	S25
S6.4 EXAFS analysis on the $Cu(II)\text{-}OO\text{-}Cu(II)]^{2+}_{endon/sideon}$ structures	S26
S7. Resonant Raman spectroscopy	S29
References	S31

S1. Experimental details

S1.1. Materials

A home-made Cu-MOR sample was synthesized according to the literature. In detail, 2.6 g of NaOH (Merck, >99%) were mixed with 120 g of H_2O. Then 1 g of $NaAlO_2$ (Sigma Aldrich, Al_2O_3 50-56%, Na_2O 37-45%) was added to the solution and stirred until complete dissolution was achieved. Finally, 30 g of colloidal silica (LUDOX® SM 30 wt.%, Sigma

Aldrich) were poured in the solution. After 2 h of stirring, the obtained white suspension was transferred to a Teflon-lined autoclave (150 ml) and crystallization was carried out at 180 °C for 67 hours in a tumbling oven. The product was recovered by filtration, dried for 2 hours, and finally calcined at 550 °C for 8 h, reached with a heating slope of 1 °C/min. The obtained Na-MOR sample (Si/Al = 8.2 by EDX) was ion exchanged to obtain it in Cu form. Initially, the Na-MOR was exchanged by a 1 M NH_4NO_3 solution at 60 °C for 5 h, in order to obtain the sample in protonic form. The exchange procedure was repeated three times, supplying fresh NH_4NO_3 solution at each stage. At the end of the third repetition, the sample was washed with abundant distilled water and then calcined at 550 °C for 8 h (ramp rate 1 °C/min). Finally, the amount of Cu(II) acetate monohydrate (Sigma-Aldrich, 99.99%) required to obtain a concentration of 2.5 mM was dissolved in water and the proton form of the zeolite was added to the solution (250 ml per zeolite gram). The mixture was stirred at 60 °C for 48 h and then the obtained copper zeolite was filtered, washed, dried at 100 °C overnight and calcined at 550 °C for 5 hours to remove the residual acetate. The sample was prepared for XAS measurements in the form of self-supporting pellets, with thickness optimized for transmission measurements. All the pellets were initially dehydrated in vacuo at 500 °C and then subjected to different treatments; one of them was cooled and stocked in this condition, to serve as a reference for self-reduction (SR). A pellet was directly exposed to pure O_2 (100 mbar) at 500 °C. Two pellets were exposed to 100 mbar of pure NH_3 at 500 °C and then outgassed; one of them was further oxidized in pure O_2 at 500 °C. Finally, four pellets were cooled down to 250°C and exposed to 100 mbar of CH_4, CO, H_2 and NH_3 respectively; these pellets were further heated to 500 °C in dynamic vacuum to remove residual gas molecules adsorbed on the

solid. All temperature ramps in the treatments were set to 5°C/min. The pellets were then transferred in a glove box to avoid contact with air, individually sealed in vacuum plastic bags, and stored in a N_2-filled canister for transportation.

The samples for resonant Raman measurement were treated in a flow (20 ml/min) of pure oxygen at 500 °C for 2 h (ramp 5 °C/min) inside a quartz capillary (1 mm inner diameter). Then, the sample was exposed to NH_3 (1800 ppm in He for 30 min, 20 ml/min) and again to O_2 for further 2 h to assess the effect of pre-reduction on the formed Cu-oxo species. After the treatment, the capillary was sealed on both ends and the sample measured at room temperature.

S1.2. Characterization

Powder XRD was collected in Bragg-Brentano geometry with a Panalytical X'pert PRO diffractometer, equipped with a Cu k_{α} X-ray source and a X'celerator strip detector. The Cu k_{β} line was filtered by a Ni foil.

N_2 adsorption/desorption isotherms at 77 K were collected with a Micromeritics ASAP 2020 automated volumetric sorption instrument. Prior to measurement, the sample was outgassed at 400 °C (reached with a 5 °C/min ramp) for 4 h, in order to remove preadsorbed molecules. Specific surface areas were determined by using both the Brunauer–Emmett–Teller (BET) and the Langmuir models. Pore size distributions were obtained by applying the Non-Localized Density Functional Theory (NL-DFT) method.

Cu K-edge XAS spectra were collected at the XAFS beamline at the ELETTRA Synchrotron (Trieste), in transmission mode using a Si(111) double-crystal monochromator. The incident (I_0) and transmitted (I_1) X-ray intensities were detected in the range within 8780 and 10500 eV (40 min/scan, 3 spectra collected per sample) using

two ionization chambers filled with a mixture of He and Ar. Finally, a third ionization chamber (I_2) was used for the simultaneous collection of a Cu metal foil XANES spectrum employed in the energy calibration procedure. The collected XAS spectra were then aligned in energy and normalized to unity edge jump using the Athena software from the Demeter package.[1] The $\chi(k)$ EXAFS signals were extracted using the same program. In particular, the Fourier transform (FT) of the EXAFS spectra were recovered by transforming the $k^2\chi(k)$ functions in the 2.4–12.4 Å^{-1} range (a Hanning-apodization window was adopted), while the corresponding Wavelet Transform representations were realized employing a Morlet mother function[2,3] with the resolution parameters σ and η set to 1 and 7, respectively. The latter were optimized to have the highest resolution in both k and R spaces and to highlight the possible presence of multi-copper moieties located within the second and third shells of the FT-EXAFS spectra.[4]

Resonant Raman spectra were collected with a Renishaw inVia Raman microscope spectrometer, equipped with a He-Cd laser (Kimmon Koha IK series) emitting at 442 nm as excitation source. The excitation light was focused on samples through a 20x objective, analyzed by a 2400 lines/mm grating and detected by a CCD detector. The laser power was set to ca. 0.1 mW at the sample, in order to avoid sample degradation induced by the radiation.

S2. Characterization of the home-made Cu-MOR samples

Figure S1 shows the powder XRD pattern and the N_2 adsorption isotherm at 77 K of the home-made Cu-MOR sample studied in this work.

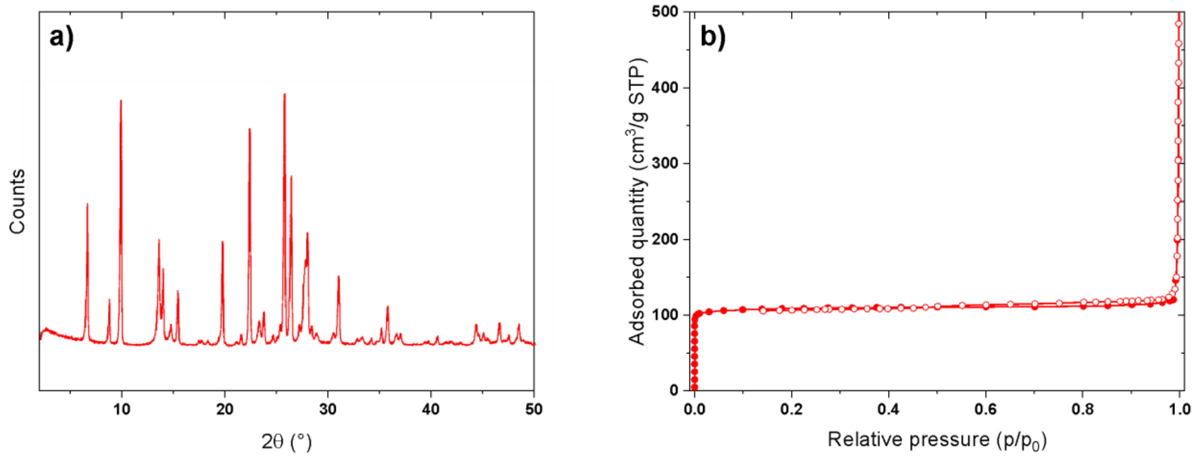

Figure S1. a) Powder XRD pattern; and b) N_2 adsorption/desorption isotherm at 77 K (full dots: adsorption, empty dots: desorption) for the home-made Cu-MOR adopted in this work.

The powder XRD pattern shows as the synthesized material is crystalline and does not contain phases other than the Mordenite one.[5] The N_2 adsorption isotherm presents the characteristic type I shape as expected for a microporous zeolite. Accordingly, the specific surface area was evaluated to be 350 m^2/g (BET model) or 480 m^2/g (Langmuir model). The micropore volume, evaluated by NL-DFT fitting of the isotherm (Oxide Cylindrical Pores Strong Potential model by Micromeritics), equals 0.14 cm^3/g and is ascribed to a monodispersed family of micropores with a diameter or ca. 8.0 Å (*i.e.* compatible with the pore diameter of the MOR framework).

Figure S2 compares the Raman spectra of the Cu-MOR sample synthesized in this work and that of the sample labeled as "0.18-7" by Pappas et al. in a previous

contribution.[6] The latter was obtained by ion exchange starting from a commercial Zeolyst CBV10A Na-MOR sample.

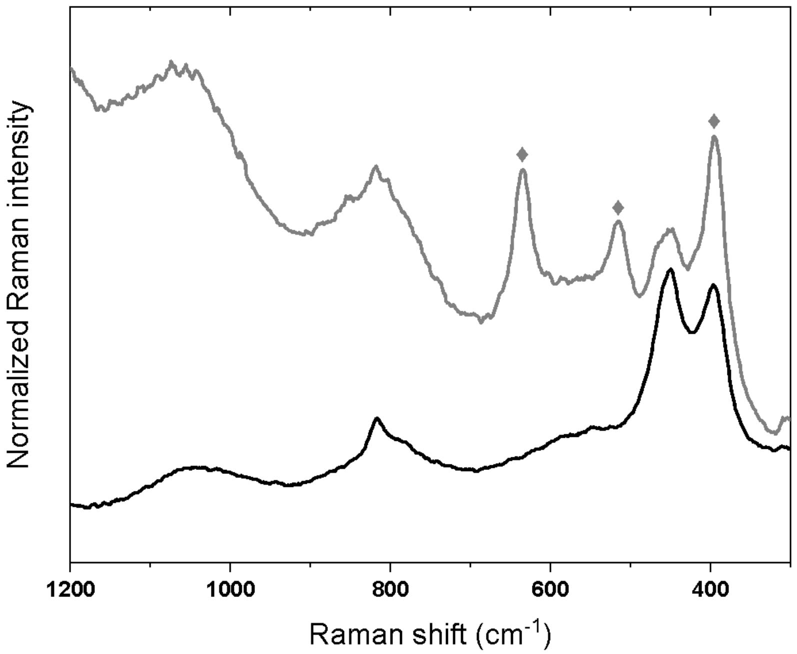

Figure S2. Raman (λ = 442 nm) spectra of Cu-MOR 0.18-7 (gray curve) and of Cu-MOR synthesized ad hoc in this work (black curve). Gray diamonds (◆) highlight the position of the features associated to TiO_2 anatase.

The 0.18-7 sample clearly exhibits a significant content of TiO_2 anatase as pollutant, the intense features of which (sharp peaks at 395, 515 and 635 cm^{-1})[7] hamper the identification of the underlying signals from the MOR framework and the possibly observed Cu-oxo species. For this reason, the sample synthesized specifically for this work has been preferred, since it allows the collection of unbiased Raman spectra.

S3. Details on XANES analysis

S3.2 Comparison of XANES spectra for selected Cu-MOR and Cu metal foil

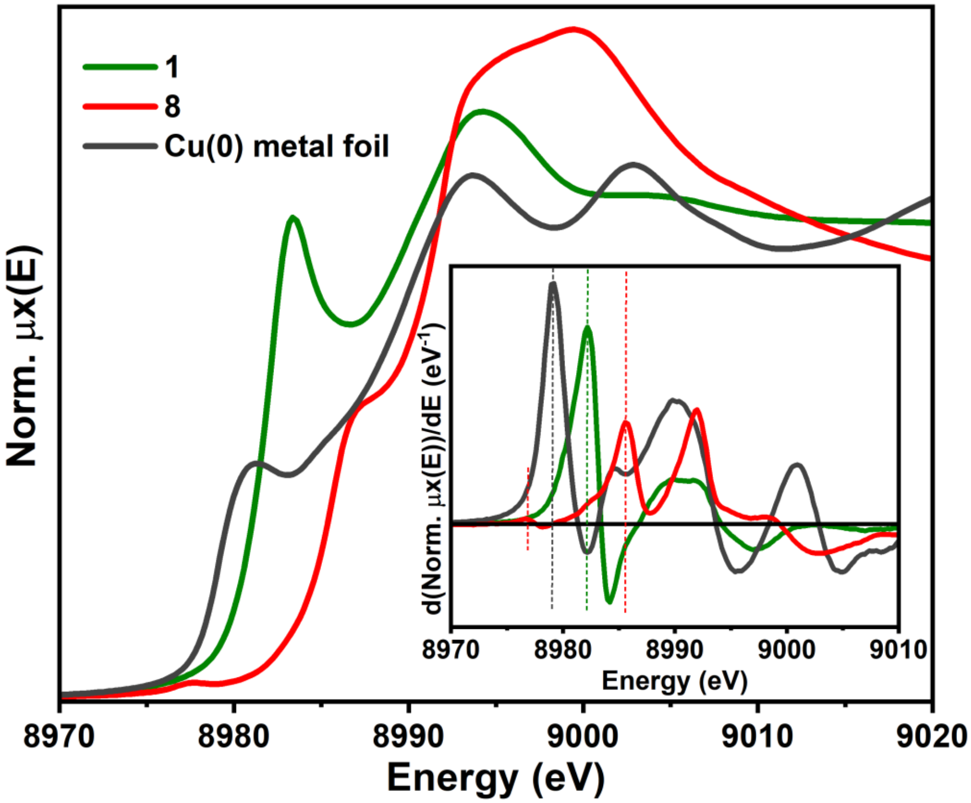

Figure S3. Comparison between XANES spectra for 'end-member' Cu-MOR samples (1 - reduced in NH_3 at 500 °C and 8 - pre-reduced in NH_3 and oxidized in O_2 at 500 °C) and reference Cu(0) metal foil. The inset report the corresponding first-derivative XANES curves, with vertical dashed lines indicating the relevant peak positions for the three samples. No detectable peaks are observed in both Cu-MOR samples at the energy characteristic of metallic Cu (ca. 8979 eV). The weak peak observed at ca. 8977 eV for sample 8 corresponds to the Cu(II) 1s->3d peak. The same observations are valid for the other Cu-MOR samples (2-7), not reported here for graphical clarity.

S3.2 PCA analysis of the XANES dataset

The first step in the analysis of the *ex situ* XAS data collected during the procedure described in the main text and here in **Section S1.1** stands in the identification of the number of pure Cu species, the combination of which is able to explain the data variances observed in the experimental spectral series. For this reason, we focused our attention on the XANES, restricting the analysis in the range within (8975-9020) eV, as reported in **Figure 1a** of the main text. We then applied Principal component analysis (PCA) to the dataset. **Figure S3a** reports the first four abstract principal components (PCs) derived through the Singular Value Decomposition (SVD)[8] of the XANES series. From a visual inspection of these profiles, it is possible to notice that only the first two abstract spectra show a series of intense features which can be properly associated to independent chemical/physical sources, while only weak and negligible variations in the intensities appear already from the third component. This evidence is also underlined looking at the trend of the singular values emerging from the Scree Plot (*i.e.* the plot of the extracted singular values *vs* the number of components involved in the reconstruction of the XANES dataset), where a well-defined elbow appears in proximity of the second principal component, justifying the presence of two main species (see **Figure S3b**).

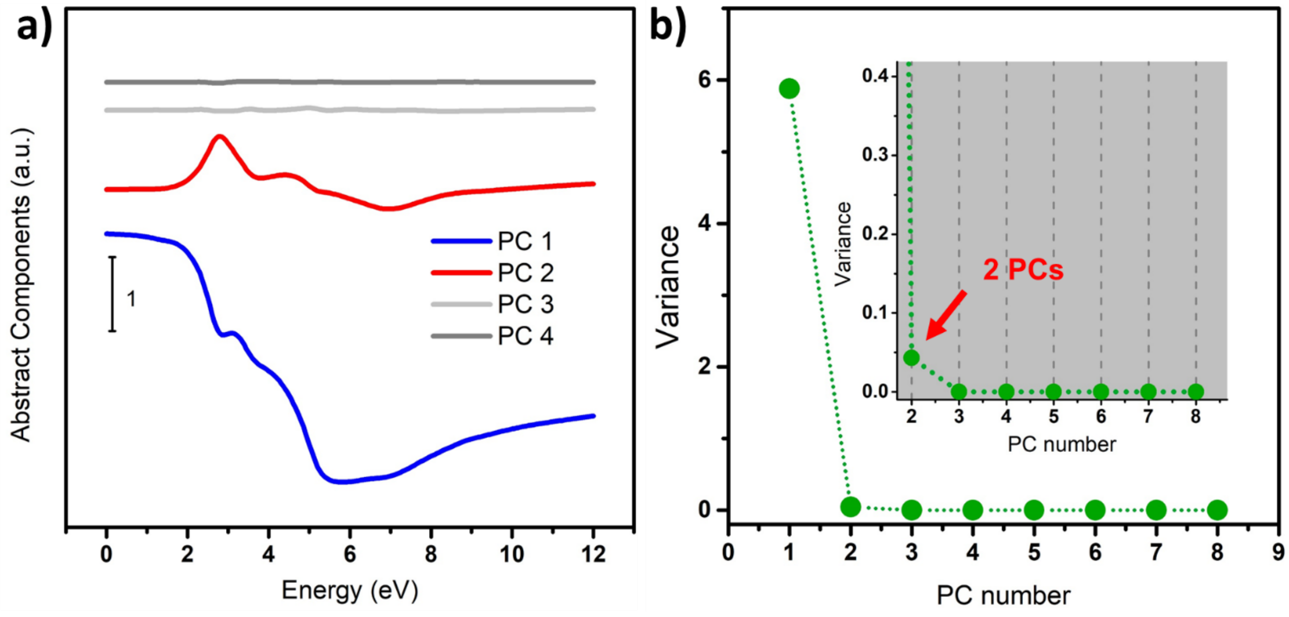

Figure S4. a) First four abstract components derived from the singular value decomposition of the XANES dataset reported in **Figure 1** of the main text. b) Trend of the variance associated to the eigenvectors of the XANES dataset covariance matrix (*i.e.* Scree plot). The inset represents a magnification of the main plot where it is possible to appreciate around the second PC the elbow in the curve.

S3.2 Comparison between experimental and LCF XANES spectra

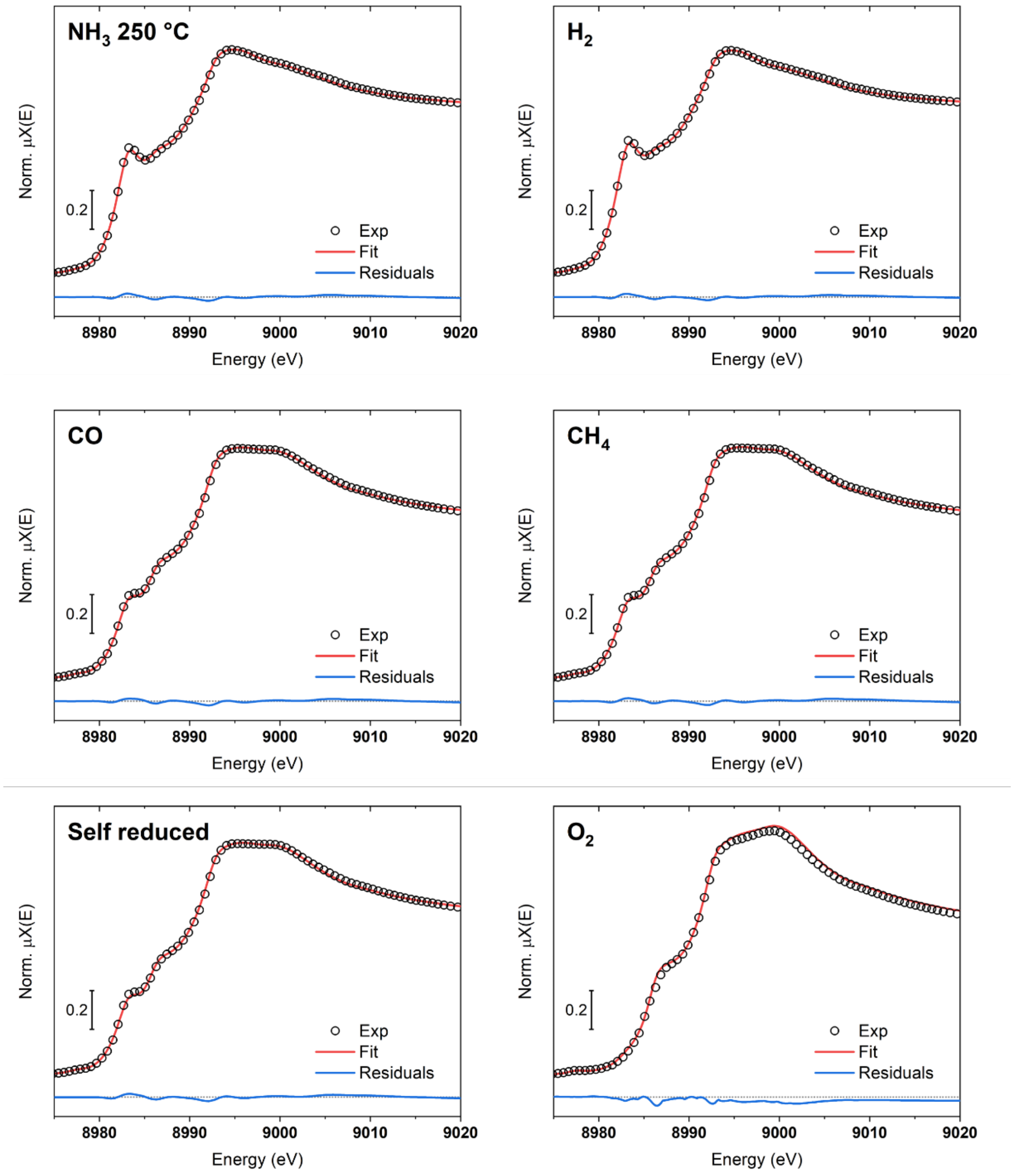

Figure S5. Comparison between experimental XANES spectra (black circles, spectra 2-6 reported in **Figure 1** of the main text, reported here visualizing one every three energy points, for graphical clarity) and correspondent best-fit curves from LCF analysis (red lines). LCF residuals are also reported as blue lines.

S4. EXAFS Wavelet Transform Analysis

S4.1. Full range wavelet transform representation

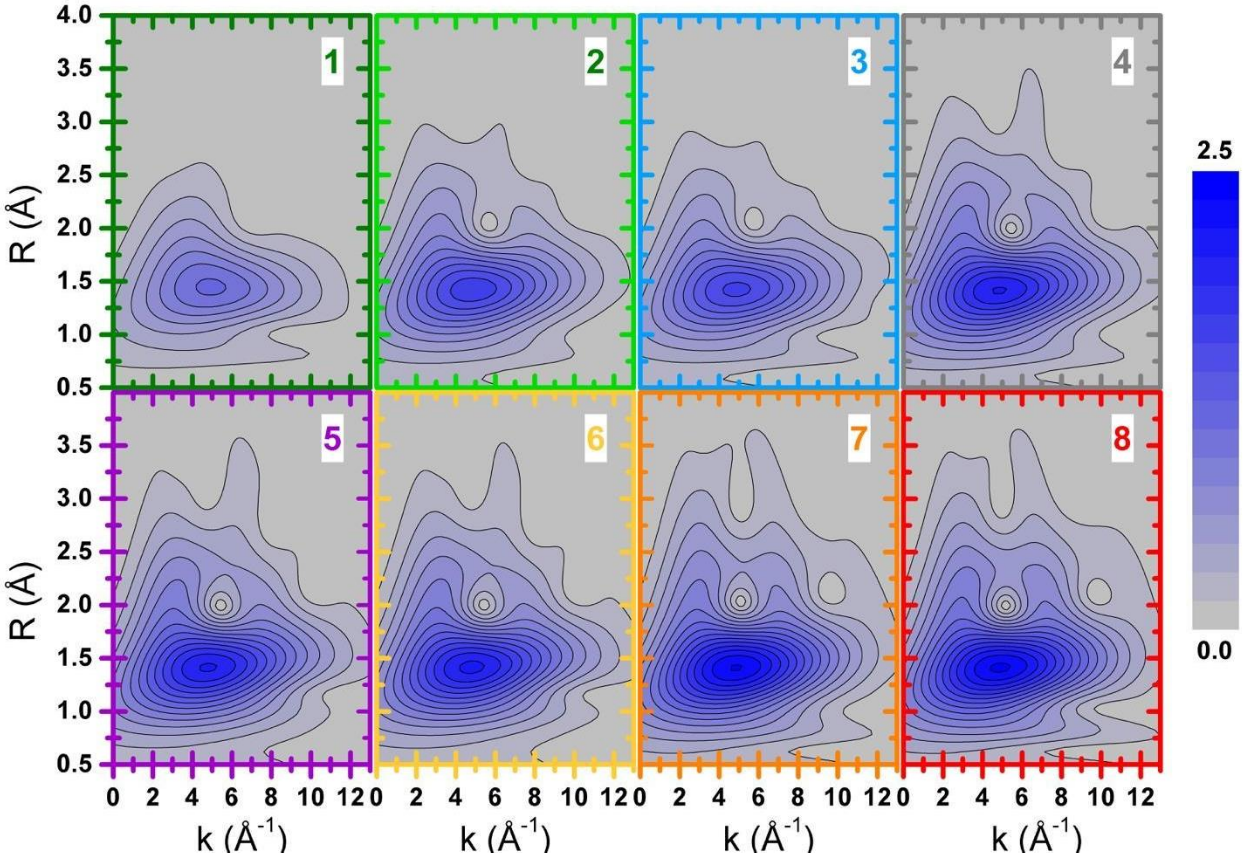

Figure S6. Full range wavelet transform representation of the EXAFS signal for Cu-MOR: 1) reduced in NH_3 at 500 °C; 2) reduced in NH_3 at 250 °C; 3) reduced in H_2 at 250 °C; 4) reduced in CO at 250 °C; 5) reduced in CH_4 at 250 °C; self-reduced by vacuum dehydration at 500 °C; 7) directly oxidized in O_2 at 500 °C; and 8) pre-reduced in NH_3 and oxidized in O_2 at 500 °C.

S4.2. Representative FEFF-derived Backscattering amplitude Factors

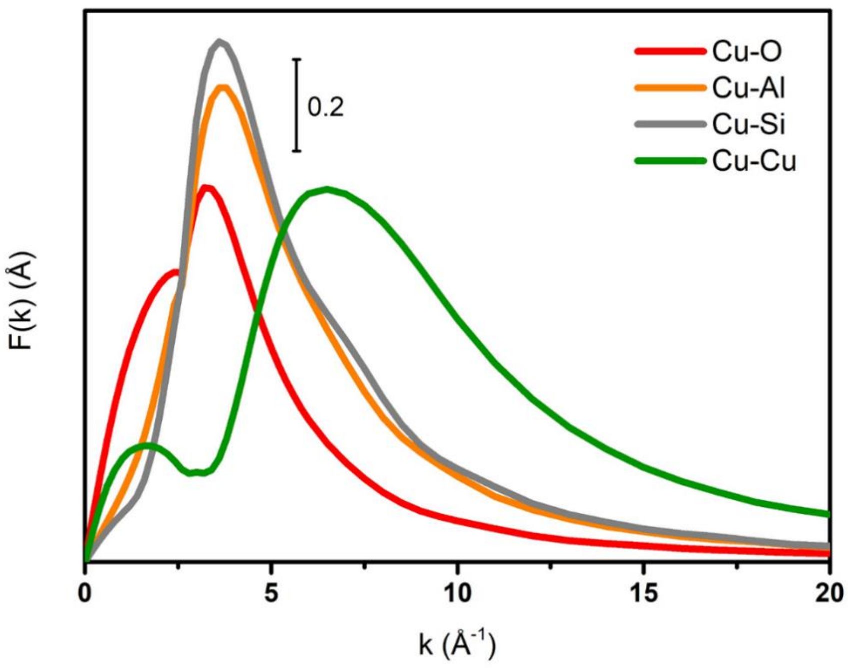

Figure S7. FEFF-derived backscattering amplitude factors F(k) extracted for the Cu-O (red profile), Cu-Si (grey profile), Cu-Al (orange profile) and Cu-Cu (green profile) single scattering contribution. The Cu mono-μ-oxo structure shown in **Figure S7b** was employed as the input for their calculation.

S5. Computational screening on Cu pairs distribution in 8-MR side pockets

The most challenging task in the computational study of Cu sites in zeolites is to account for the cation distribution within the zeolite framework. This is strictly related to the Al distribution and by the overall composition (Si/Al and Cu/Al). A systematic approach to the problem requires to explicitly compute all the possible configurations for Si substitution by Al, to sort them based on their relative stability and, as a second stage, to select which of them are the most energetically favored upon Cu exchange. In this work, we specifically considered Al substitution within the 8-MR side pocket of the MOR structure through a cluster approach. We initially optimized the fully periodic structure of the purely siliceous MOR at the B3LYP/def2-SVP level of theory with the CRYSTAL17 code.[9] An optimized set of computational parameters, already successfully adopted in the simulation of CHA[10] and MFI[11] frameworks, was adopted. The side pocket cluster was cut from the relaxed periodic structure by truncating Si-O bonds and by saturating the missing atoms with Si-H, positioned along the direction of the pristine Si-O they replaced at fixed distance (1.45 Å). Such structural models feature 185 atoms (including 42 H atoms as terminations, was kept frozen during the subsequent optimization) and contain 20 tetrahedral sites (side pocket excluded). The structure of this cluster, showing all the possible substitutional sites, is presented in **Figure S8**.

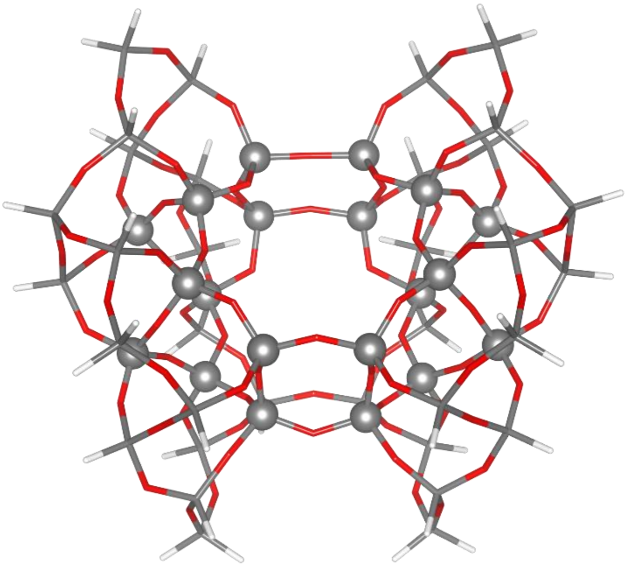

Figure S8. Purely siliceous cluster model of MOR side pocket. Spheres represent Si atoms included in the combinatory study for 2 Al substitution. Other atoms are represented with bonds only. Atoms color code: Si, grey; O, red; H, white.

From this purely siliceous cluster, 162 structures containing 2 Al atoms (complying with Löwenstein's rule) were generated. A Na^+ cation per Al atom was inserted to keep the overall charge neutrality. Due to the high number of involved structures, a DFT approach is not easily suitable because of the excessive computational cost. Accordingly, a tight binding approach was adopted in the structural screening stage, taking advantage of the GFN2-xTB[12] method as implemented in the xTB (version 6.3.1).[13] By adopting this method, the structure of the 162 models were optimized and ranked based on their energy. We also estimated the probability p_i of having a defined structure i on the basis of the Boltzmann distribution:

$$p_i = \frac{e^{-\frac{\Delta E_i}{k_b T}}}{\sum_i \ e^{-\frac{\Delta E_i}{k_b T}}}$$

where k_b is the Boltzmann constant, T is the temperature (set to 25 °C) and ΔE_i is the relative stability of the ith structure with respect to the most stable one, i.e. defined as

$$\Delta E_i = E_i - \{E_i\}$$

The population analysis showed as only 13 structures have, cumulatively, more than 80% probability of existing. Accordingly, Na^+ substitution by Cu^+ was explored for this subset of models. The 13 Cu-containing structures were again optimized and ranked on their relative energy/Boltzmann probability. Finally, the most favorable model in terms of both Al distribution and Cu^+ exchangeability was determined by multiplying the probabilities obtained after the two stages described above. The key results are reported in **Table S1**. Finally, the model labeled as 13-19 (shown in **Figure S9a** in its $2[Cu(I)]^+$ form) was selected as the most reliable structure to proceed onto the study on Cu-oxo species with a more rigorous DFT approach. In detail, starting from the $2[Cu(I)]^+$ model, three Cu-oxo structures were generated: a mono-μ-oxo dicopper site ($[Cu(II)\text{-}O\text{-}Cu(II)]^{2+}$) and two peroxo dicopper sites, with the oxygen coordinated respectively with a side-on ($[Cu(II)\text{-}OO\text{-}Cu(II)]^{2+}{}_{sideon}$) or end-on ($[Cu(II)\text{-}OO\text{-}Cu(II)]^{2+}{}_{endon}$) likely geometry. All the DFT models are graphically represented in **Figure S9**. The DFT calculations were performed with the ORCA (version 4.2.1) package,[14] by exploiting the B3LYP hybrid GGA functional[15] and the Ahlrichs def2-SVP basis set on all atoms.[16] Due to the size of the cluster, the RIJCOSX approximation strategy for Coulomb and Hartree-Fock exchange integrals was adopted,[17] relying on the def2/j as auxiliary basis set.[18]

Table S1. Probabilities for having a given 2Al distribution, for having Cu^{+} exchange on the same structures and their combined value. The difference in energy ΔE, calculated with respect to the most stable site, is reported in kJ/mol.

Model label	ΔE 2Al	p_i 2Al	ΔE Cu^{+}	p_i Cu^{+}	Combined p_i
13-19	3.9	0.02978	0.0	0.53624	0.01597
1-13	1.8	0.07047	2.7	0.18112	0.01276
8-17	3.1	0.04229	4.1	0.10453	0.00442
3-16	2.7	0.04903	4.7	0.07922	0.00388
6-19	3.3	0.03766	5.0	0.07102	0.00267
2-5	2.4	0.05435	9.9	0.00975	0.00053
4-7	2.2	0.06041	10.4	0.00810	0.00049
7-18	0.3	0.13123	11.3	0.00191	0.00025
15-18	3.5	0.03540	14.0	0.00560	0.00020
5-20	0.0	0.14524	15.4	0.00108	0.00016
4-15	1.6	0.07738	16.2	0.00078	0.00006
2-12	3.1	0.04231	16.6	0.00065	0.00003

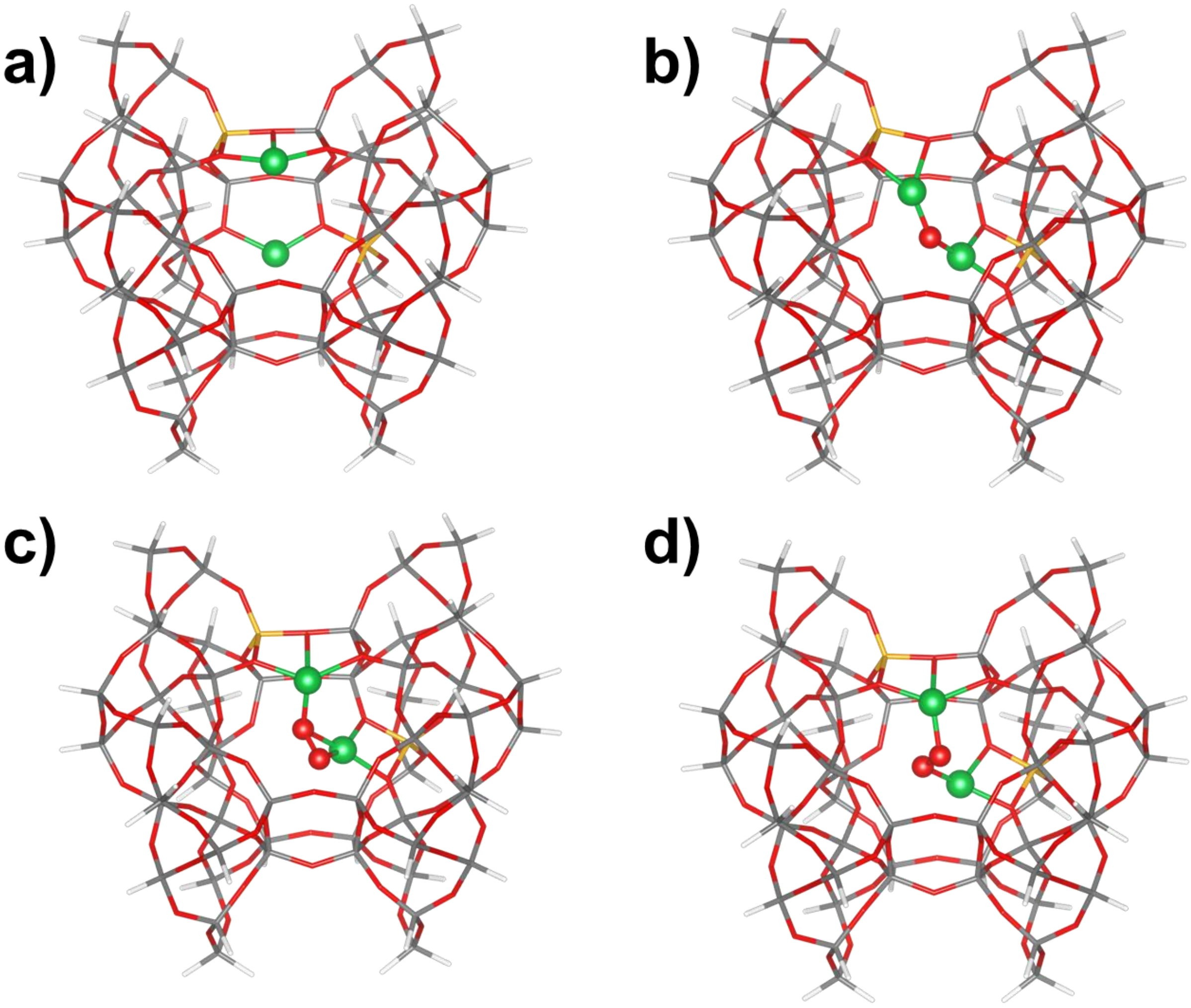

Figure S9. DFT optimized structural models for: a) 2[Cu(I)]$^{+}$; b) [Cu(II)-O-Cu(II)]^{2}; c) [Cu(II)-OO-Cu(II)]$^{2+}_{sideon}$; and d) [Cu(II)-OO-Cu(II)]$^{2+}_{endon}$. Atoms color code: Cu, green; Si, grey; Al, yellow; O, red; H, white.

S6. EXAFS analysis

S6.1 Fitting strategy

All the reported EXAFS fits were carried out in R-space, within the 1.0–3.4 Å range, employing the Artemis software from the Demeter package[1] on the k^2-weighted EXAFS spectra, Fourier-transformed in the 2.4–12.5 $Å^{-1}$ k-range. For each scattering path, the amplitudes and phases were calculated using the DFT-optimized models of the relevant Cu sites, depending on the selected experimental conditions. In particular, the EXAFS fits the spectra collected for the sample treated at 500 °C in NH_3 (sample 1 in **Figure 1**, main text); and further oxidized in pure O_2 at 500 °C (sample 8 in **Figure 1**, main text) were performed starting from the guessed structural models referring to the $2[Cu(I)]^+$ and the $[Cu(II)\text{-}O\text{-}Cu(II)]^{2+}$ structures (see **Figure S9**), respectively.

In all the analyzed cases, in order to limit the number of optimized variables, all the included scattering paths were parameterized with the same guessed passive electron reduction factor S_0^2 and energy shift term (ΔE), in accordance with our previous works.[4,6,19–21]

Because two absorbers are present for both the cases, we proceeded to consider them as two separate sites running the FEFF6.0l code[22] for each of them and extracting the relative most intense paths up to a maximum distance of 4.0 Å. This implied to write the total EXAFS theoretical equations as the average of the contributions stemming (for each case) from all the involved Cu sites as:

$$\chi_{Theory}^{TOT}(k) = 0.5 \cdot (\chi_{Cu_1}(k) + \chi_{Cu_2}(k))$$

Where $\chi_{Cu_{1,2}}(k) = \sum_i \chi_i(k)$, while $\chi_i(k)$ are the single paths involved in the reconstruction of the total EXAFS theoretical signal.

For both fitting procedures, we included the single-scattering (SS) paths proper of the first-shell O atoms of the zeolite framework (O_{fw1}), as well as those contributions deriving from the second oxygen atoms sited closer to the Cu absorber, sited inside (O_{fw2}) or outside (O_{efw}) the zeolite framework. The latter, in particular, are specific for the $[Cu(II)-O-Cu(II)]^{2+}$ structure. A second shell of framework Si/Al atoms (T_{fw}) was included for both the models too.

All the SS paths have been parameterized with independent interatomic distances and Debye-Waller (DW) factors for each coordination shell. It is worth noting that, we found suitable to describe the first and second oxygen shells of the model referring to the NH_3 reduced state with two distinct DW parameters: σ^2_{O1} and σ^2_{O2}. On the other hand, just one DW term (i.e. σ^2_{O1}) was found necessary to take account for the mean square displacements of the oxygen atoms (framework and extra framework) for the 500 °C oxidized sample. Below the second shell, the SS paths stemming from farer Si and O atoms of the framework (fw) up to 3.4 Å were included and parameterized by means of a global contraction/expansion factor α_{fw} and a DW factor σ^2_{fw} increasing as the square root of the distance $R_{eff,i}$ of the ith scattering atom from the absorber ($\Delta R_{fw,i} = \alpha_{fw}\, R_{eff,i}$, $\sigma^2_{fw,i} = ss^2_{fw}\, (R_{eff,i}/R_0)^{1/2}$, where R_0 indicates the shortest R_{eff} for the group of paths). Finally, the contribution deriving from the Cu-Cu interaction was accounted only for the Cu(II) mono-μ-oxo structure by including a $Cu-Cu_{ef}$ SS path with the initial DFT distance set to ca. $R_{eff} = 3.31$ Å. Herein the related SS contribution was refined as a guess interatomic distance R_{Cu} possessing a Debye-Waller factor σ^2_{Cu}. Differently from the $[Cu(II)-O-Cu(II)]^{2+}$ case,

in the $2[Cu(I)]^+$ structure, the SS path involving the Cu-Cu interaction was not added for the fitting procedure since the interatomic distance between the two Cu atoms was found to be higher than 5 Å from the DFT. In particular, the half path length of this interaction falls in an R region characterized by weak oscillations comparable to the ripple deriving from the noise contribution and completely outside of the selected fitting range.

S6.2 EXAFS best fits

The fit results as well as the EXAFS best-fit curves, for both the structures, are reported in **Table S2, S3, S4** and **Figure 4** of the main text. It is worth noting that, for both the analyzed geometries, the SS paths signals describing the same type of scattering process but belonging to different Cu absorbers have been averaged (e.g. $Cu_1 \rightarrow O_{fw1}$ and $Cu_2 \rightarrow O_{fw1}$ have been reported as $< O_{fw1}>$). For the sake of clarity all the EXAFS paths curves are reported in the following figures as not averaged contributions for both the analyzed structures. Although comparable with 1 within its uncertainty, the S_0^2 term appears to be slightly large. Based on this observation and in line with the heterogeneity of the Cu sub-lobe observed in the WT map, we cannot exclude the presence of minor fractions of different Cu^{2+} species with a higher coordination number in the first shell. Inclusion of such contributions in the fitting model would plausibly lead to a decrease of the S_0^2 term, but to the expense of fitting reliability due to the necessary increase in the number of fitting parameters. Nevertheless, the retrieved parameters are in accordance with the DFT-optimized $[Cu(II)\text{-}O\text{-}Cu(II)]^{2+}$ model used as a starting point for the refinement. Conversely, if the higher-coordination case described by the $[Cu(II)\text{-}O\text{-}O\text{-}Cu(II)]^{2+}_{side\text{-}on}$ model is solely considered, a similar fitting quality is not found (see section S6.4) confirming the reliability of our results.

Table S2. Results of the fits performed on the k^2-weighted FT-EXAFS spectra referring to the MOR samples reduced at 500 °C in NH_3 (sample 1 in **Figure 1**) and further oxidized in pure O_2 at 500 °C (sample 8 in **Figure 1**). The fitting range was 1.0-3.4 Å for both the analyzed cases. The the symbol < > denotes the averaged distances refined by the fit corresponding to a series of contributions defining a specific EXAFS coordination shell. The $2[Cu(I)]^+$ and the $[Cu(II)\text{-}O\text{-}Cu(II)]^{2+}$ structures (**Figure S9a** and **S9b**, respectively) were adopted as structural model in the fit of Sample 1 and Sample 8, respectively.

EXAFS parameters	Sample 1	Sample 8
$N°_{par}/N°_{ind}$	10/15	11/15
$\%R_{factor}$	0.8	0.3
S_0^2	0.9±0.1	1.1±0.1
ΔE (eV)	-7±2	-2.2±0.9
$<R_{Oefw}>$(Å)	-	1.97±0.02
$<R_{Ofw1}>$ (Å)	1.93±0.01	1.93±0.02
$<R_{Ofw2}>$ (Å)	2.64± 0.09	-
$<R_{Tfw}>$ (Å)	2.94±0.03	2.68±0.01
α_{fw}	- 0.08±0.01	0.00±0.02
$R_{Cu\text{-}Cu}$ (Å)	-	3.28±0.08
σ^2_{O1} (Å^2)	0.005±0.001	0.002±0.001
σ^2_{O2} (Å^2)	0.02±0.02	-
σ^2_{Tfw} (Å^2)	0.013±0.007	0.004±0.002
ss^2_{fw} (Å^2)	0.02±0.01	0.019±0.009
σ^2_{Cu} (Å^2)	-	0.016±0.008

Table S3: Coordination numbers of the identified shells contributing to the fit with the $2[Cu(I)]^{+}$ model.

Shell	Site 1	Site 2
O_{fw1}	2	2
O_{fw2}	1	1
T_{fw}	2	0
fw	3	9

Table S4: Coordination numbers of the identified shells contributing to the fit with the $[Cu(II)\text{-}O\text{-}Cu(II)]^{2+}$model.

Shell	Site 1	Site 2
O_{efw}	1	1
O_{fw1}	2	2
T_{fw}	1	1
fw	5	3

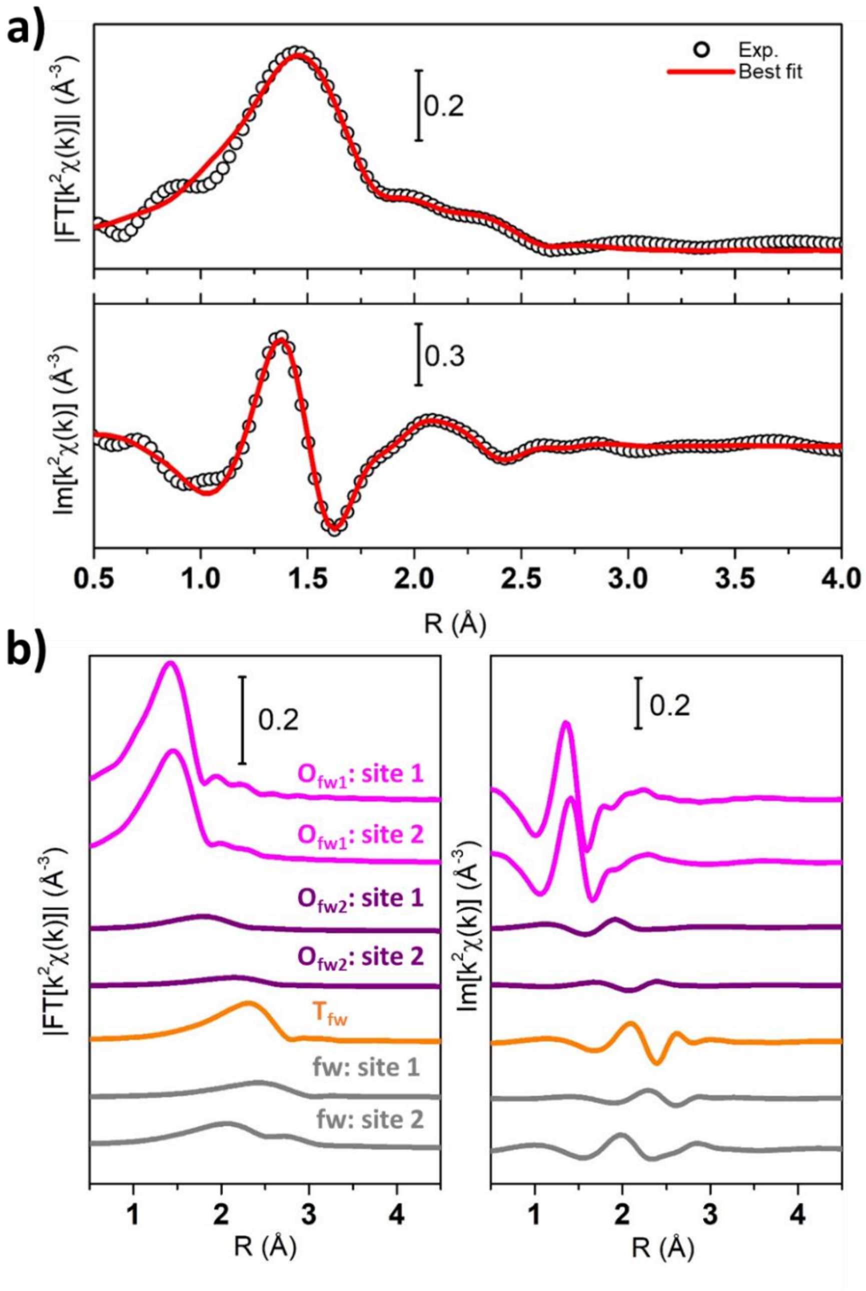

Figure S10. a) Phase uncorrected modulus (top) and imaginary part (bottom) of the experimental and best fit FT EXAFS spectra referring to the NH_3 treated sample at 500 °C, realized using the $2[Cu(I)]^+$ structure (**Figure S9a**). The experimental data are shown as black open circles and the best fits with red solid lines. The principal SS contributions included in the fitting model b) are reported as colored solid lines grouped as a function of the scattering type (see **Figure 4a'** in the main text) and vertically for sake of clarity.

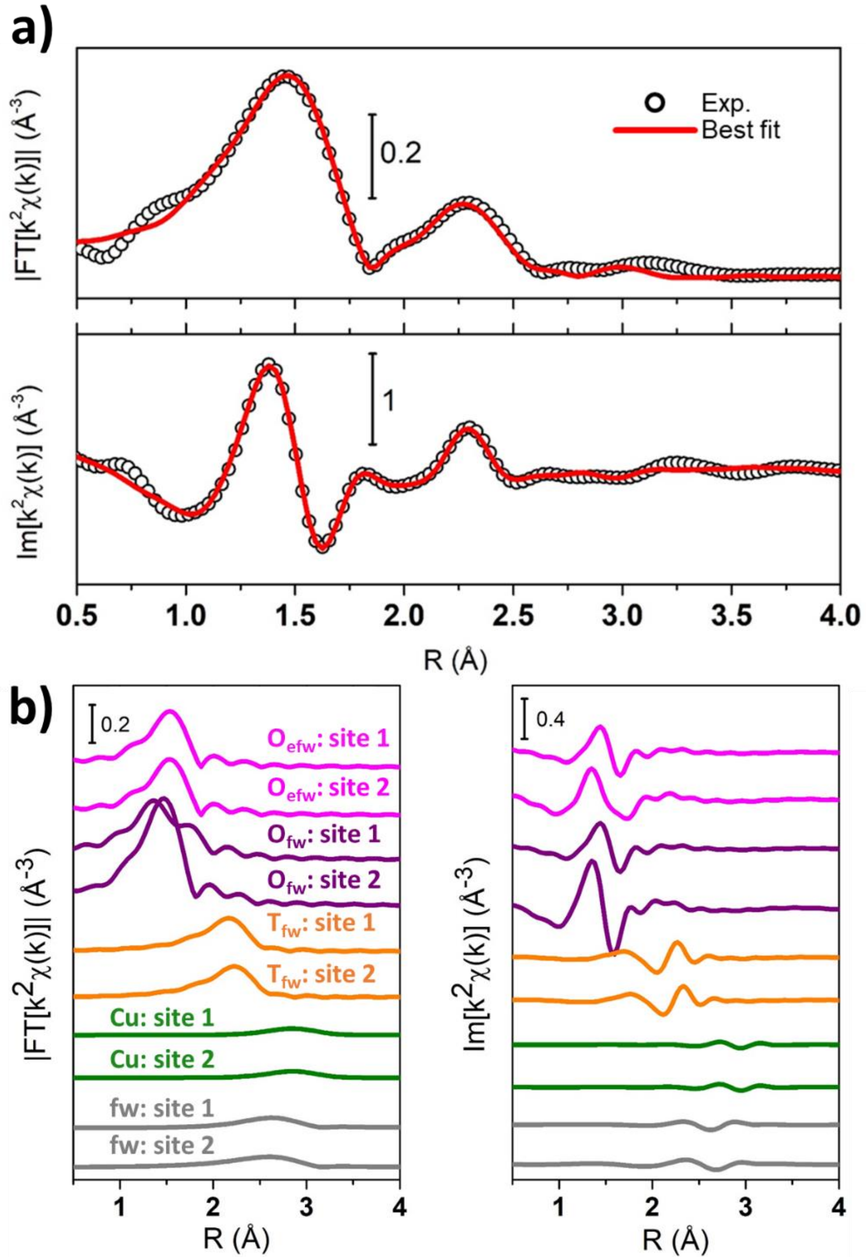

Figure S11. a) Phase uncorrected modulus (top) and imaginary part (bottom) of the experimental and best fit FT EXAFS spectra referring to the O_2 treated sample at 500 °C, realized using the $[Cu(II)\text{-}O\text{-}Cu(II)]^{2+}$ structure (**Figure S9b**). The experimental data are shown as black open circles and the best fits with red solid lines. The principal SS contributions included in the fitting model b) are reported as colored solid lines grouped as a function of the scattering type (see **Figure 4b'** in the main text) and vertically for sake of clarity.

S6.3 Experimental and best-fit EXAFS spectra in k-space

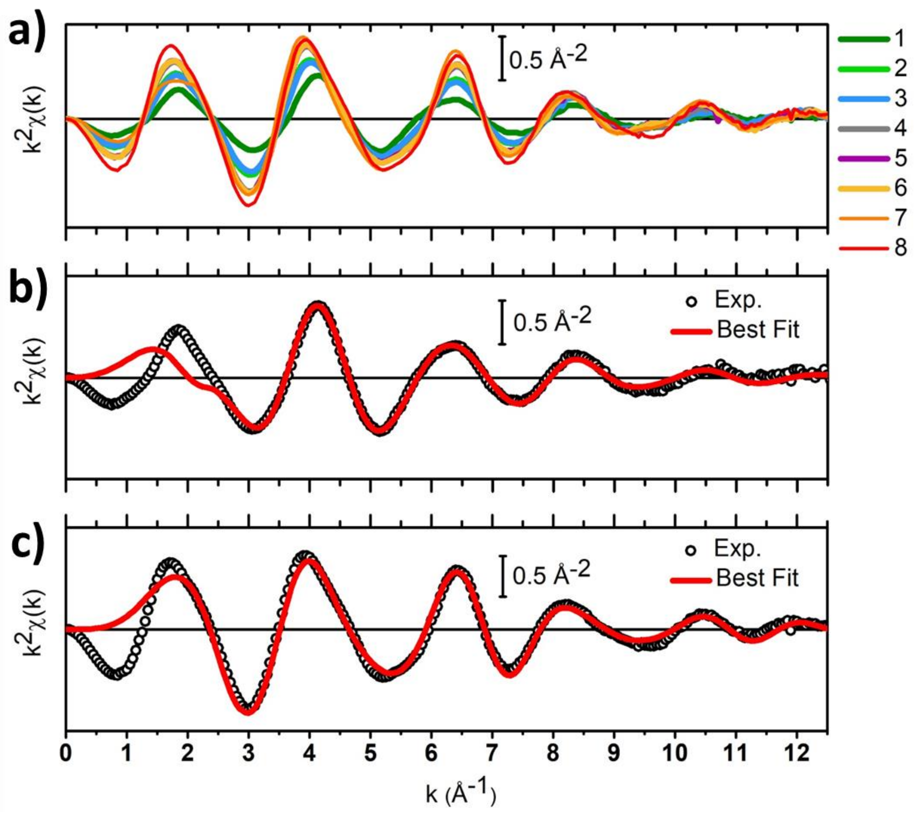

Figure S12. a) Raw $k^2\chi(k)$ EXAFS spectra for Cu-MOR: 1) reduced in NH_3 at 500 °C; 2) reduced in NH_3 at 250 °C; 3) reduced in H_2 at 250 °C; 4) reduced in CO at 250 °C; 5) reduced in CH_4 at 250 °C; 6) self-reduced by vacuum dehydration at 500 °C; 7) directly oxidized in O_2 at 500 °C; and 8) pre-reduced in NH_3 and oxidized in O_2 at 500 °C. b, c) Comparison between k^2 $\chi(k)$ experimental and best fit EXAFS spectra (realized in the R-space) referring to b) the NH_3 treated sample at 500 °C, realized using the $2[Cu(I)]^+$ structure (**Figure S9a**) and c) the O_2 treated sample at 500 °C, realized using the $[Cu(II)\text{-}O\text{-}Cu(II)]^{2+}$ structure (**Figure S9b**).

S6.4 EXAFS analysis on the $Cu(II)-OO-Cu(II)]^{2+}_{endon/sideon}$ structures

Starting from the [Cu(II)-OO-Cu(II)]2+endon structure, it is possible to note that the relative Cu-Cu distance is optimized by the DFT to ca. 4.0 Å. Looking at this region in the FT space (i.e. ca. 3.6 Å in the phase uncorrected space) it is possible to notice the absence of any particularly relevant physical feature. This fact is also reinforced by the WT representation, which shows only a weak ridge emerging from the FT ripple deriving mostly from the noise. Focusing on the $[Cu(II)-OO-Cu(II)]^{2+}_{sideon}$ model, the DFT-derived Cu-Cu distance from DFT is 3.57 Å, compatible with the lobe appearing in the WT representation. Accordingly, we performed the EXAFS fit in the R-space, employing this structural model and using the same fitting range as for the fit described in **Figures 4b and S11**. The model parameterization followed the one adopted for the $[Cu(II)-O-Cu(II)]^{2+}$ site as well. The results of the fitting procedure are reported in **Table S5** and **S6**.

Table S5. Results of the fits performed on the k^2-weighted FT-EXAFS spectra referring to the MOR samples oxidized in pure O2 at 500 °C (sample 8 in **Figure 1**). The fit was realized in the R space within 1.0-3.4 Å. The symbol < > denotes the averaged distances refined by the fit corresponding to a series of contributions defining a specific EXAFS coordination shell. The unphysical best-fit parameters are reported in red while the initial guessed Cu-Cu DFT optimized distance in blue inside the brackets. The $[Cu(II)\text{-}OO\text{-}Cu(II)]^{2+}_{sideon}$ structure (Figure S9c) was adopted as structural model in the fit.

EXAFS parameters	Sample 8
$N°_{par}/N°_{ind}$	11/15
$\%R_{factor}$	0.21
S_0^2	**1.26±0.07**
ΔE (eV)	-0.4±0.6
$\langle R_{Oefw} \rangle$(Å)	2.12±0.01
$\langle R_{Ofw1} \rangle$ (Å)	2.11±0.01
$\langle R_{Tfw} \rangle$ (Å)	2.74±0.01
α_{fw}	-0.06±0.02
R_{Cu-Cu} (Å) (**3.57 Å**)	**3.79±0.03**
σ^2_{O1} (Å^2)	0.001±0.001
σ^2_{Tfw} (Å^2)	0.006±0.002
ss^2_{fw} (Å^2)	**0.04±0.01**
σ^2_{Cu} (Å^2)	0.011±0.004

Table S6: Coordination numbers of the identified shells contributing to the fit with the $[Cu(II)\text{-}O\text{-}O\text{-}Cu(II)]^2_{side\ on}$ model.

**Shell**	**Site 1**	**Site 2**
O_{efw}	2	2
O_{fw1}	3	2
T_{fw}	1	1
fw	6	5

From the retrieved best-fit parameters, it is possible to note that there are some unphysical results. First of all, the variation of the Cu-Cu distance is refined to +0.23 Å, shifting the Cu-Cu contribution outside the fitting range. This quantity is quite large to be properly accounted by the Artemis based fit, which can accommodate, in a correct way, only small displacements of the atoms from their original guessed position, as discussed in detail in ref. 23.

Second, the S_0^2 factor is found to be equal to 1.26±0.07. This is an inconsistent value, since it should be always lower than 1 or comparable with the unit value within the uncertainty range.[24] Finally, the common framework ss^2_{fw} term is found to be equal to 0.04 $Å^{-2}$; a quite large quantity remembering that the XAS measurements have been collected at RT and deviating from our previous findings using similar framework models.[4,19-21]

S7. Resonant Raman spectroscopy

The resonant Raman spectrum of sample 8 (pre-reduced in NH_3 and then oxidized in O_2 at 500 °C, see **Figure 1** of the main text) is shown in **Figure S13** and compared to that of the pristine Cu-MOR in its hydrated form. The spectrum of the hydrated material closely resembles that of other cationic forms of Mordenite,[25,26] without a specific contribution from the Cu ions. In detail, the sharp peaks at 400 and 450 cm^{-1} are assigned to the framework vibrations involving 5- and 4-membered rings, respectively. The broader features at ca. 800 and 1100 cm^{-1} are instead ascribed to the symmetric and antisymmetric T-O-T stretching modes, as commonly observed for zeolites and other silicates.[25-28] Upon pre-reduction and oxidation at 500 °C, the pattern of bands in the Raman spectrum is deeply altered. In the low frequency region, the only framework signal still observable is that peaked at 400 cm^{-1} (adopted for spectral normalization), whereas the 450 cm^{-1} band is overshadowed by a doublet of broad/structured signals exhibiting their maxima at 470 and 535 cm^{-1}. The presence of high frequency shoulders can be inferred for both the bands. At high frequency, the 800 cm^{-1} band significantly broadens and shows additional features on its upper bound, namely a shoulder at around 850 cm^{-1} and a distinct peak at 875 cm^{-1}. According to the careful Raman study by Vanelderen et al.[29] such a pattern of bands is assigned to the formation of two distinct families of mono-μ-oxo dicopper sites, most probably located in the 8-memebered rings constituting the side pocket typical of the MOR framework. Such a direct observation of the final Cu-oxo species fully supports the outcomes of our EXAFS fitting procedure, the small imperfections of which (in the case of the oxidized sample) are ascribable to the heterogeneity of mono-μ-oxo dicopper sites confirmed by resonant Raman.

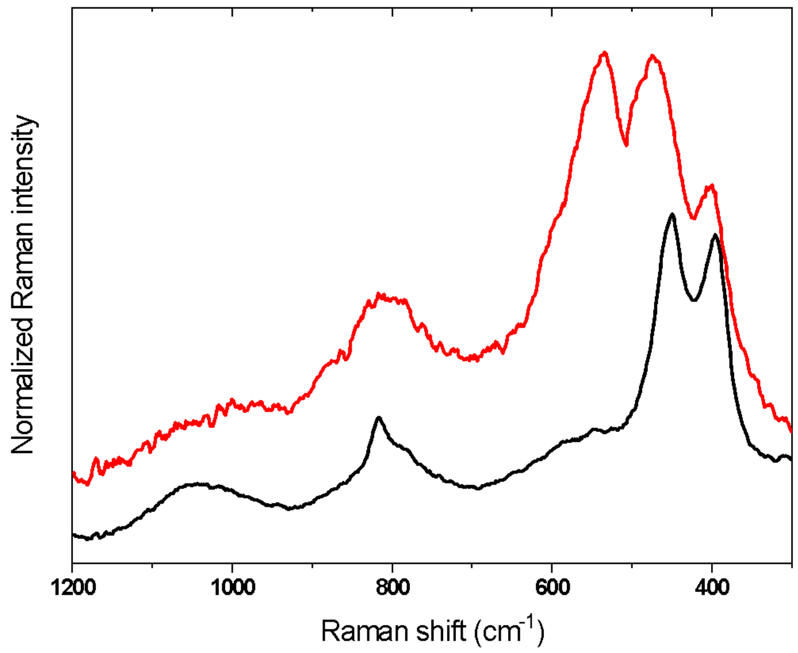

Figure S13. Resonant Raman (λ = 442 nm) of Cu-MOR on its hydrated form (black) and oxidized at 500 °C after being pre-reduced in NH_3 at 500 °C (red curve). The spectra have been normalized to the vibrational mode of the MOR framework at 400 cm^{-1}. Both spectra have been collected at room temperature, with samples sealed in quartz capillaries.

References

(1) Ravel, B.; Newville, M. ATHENA, ARTEMIS, HEPHAESTUS: Data Analysis for X-Ray Absorption Spectroscopy Using IFEFFIT. *J. Synchrotron Radiat.* **2005**, *12* (4), 537–541. https://doi.org/10.1107/S0909049505012719.

(2) Funke, H.; Scheinost, A. C.; Chukalina, M. Wavelet Analysis of Extended X-Ray Absorption Fine Structure Data. *Phys. Rev. B* **2005**, *71* (9), 94110. https://doi.org/10.1103/PhysRevB.71.094110.

(3) Timoshenko, J.; Kuzmin, A. Wavelet Data Analysis of EXAFS Spectra. *Comput. Phys. Commun.* **2009**, *180* (6), 920–925. https://doi.org/https://doi.org/10.1016/j.cpc.2008.12.020.

(4) Martini, A.; Signorile, M.; Negri, C.; Kvande, K.; Lomachenko, K. A.; Svelle, S.; Beato, P.; Berlier, G.; Borfecchia, E.; Bordiga, S. EXAFS Wavelet Transform Analysis of Cu-MOR Zeolites for the Direct Methane to Methanol Conversion. *Phys. Chem. Chem. Phys.* **2020**, *22* (34), 18950–18963. https://doi.org/10.1039/D0CP01257B.

(5) Kim, G. J.; Ahn, W. S. Direct Synthesis and Characterization of High-SiO_2-Content Mordenites. *Zeolites* **1991**, *11* (7), 745–750. https://doi.org/https://doi.org/10.1016/S0144-2449(05)80183-6.

(6) Pappas, D. K.; Martini, A.; Dyballa, M.; Kvande, K.; Teketel, S.; Lomachenko, K. A.; Baran, R.; Glatzel, P.; Arstad, B.; Berlier, G.; Lamberti, C.; Bordiga, S.; Olsbye, U.; Svelle, S.; Beato, P.; Borfecchia, E. The Nuclearity of the Active Site for Methane

to Methanol Conversion in Cu-Mordenite: A Quantitative Assessment. *J. Am. Chem. Soc.* **2018**, *140* (45), 15270–15278. https://doi.org/10.1021/jacs.8b08071.

(7) Ohsaka, T.; Izumi, F.; Fujiki, Y. Raman Spectrum of Anatase, TiO_2. *J. Raman Spectrosc.* **1978**, *7* (6), 321–324. https://doi.org/https://doi.org/10.1002/jrs.1250070606.

(8) Martini, A.; Guda, S. A.; Guda, A. A.; Smolentsev, G.; Algasov, A.; Usoltsev, O.; Soldatov, M. A.; Bugaev, A.; Rusalev, Y.; Lamberti, C.; Soldatov, A. V. PyFitit: The Software for Quantitative Analysis of XANES Spectra Using Machine-Learning Algorithms. *Comput. Phys. Commun.* **2020**, *250*, 107064. https://doi.org/https://doi.org/10.1016/j.cpc.2019.107064.

(9) Dovesi, R.; Erba, A.; Orlando, R.; Zicovich-Wilson, C. M.; Civalleri, B.; Maschio, L.; Rérat, M.; Casassa, S.; Baima, J.; Salustro, S.; Kirtman, B. Quantum-Mechanical Condensed Matter Simulations with CRYSTAL. *WIREs Comput. Mol. Sci.* **2018**, *8* (4), e1360. https://doi.org/https://doi.org/10.1002/wcms.1360.

(10) Signorile, M.; Damin, A.; Bonino, F.; Crocellà, V.; Lamberti, C.; Bordiga, S. The Role of Dispersive Forces Determining the Energetics of Adsorption in Ti Zeolites. *J. Comput. Chem.* **2016**, *37* (30), 2659–2666. https://doi.org/https://doi.org/10.1002/jcc.24509.

(11) Morra, E.; Signorile, M.; Salvadori, E.; Bordiga, S.; Giamello, E.; Chiesa, M. Nature and Topology of Metal–Oxygen Binding Sites in Zeolite Materials: ^{17}O High-Resolution EPR Spectroscopy of Metal-Loaded ZSM-5. *Angew. Chemie Int. Ed.* **2019**, *58* (36), 12398–12403.

https://doi.org/https://doi.org/10.1002/anie.201906488.

(12) C. Bannwarth, S. Ehlert and S. Grimme., *J. Chem. Theory Comput*., **2019**, *15*, 1652-1671. https://doi.org/https://doi.org/10.1021/acs.jctc.8b01176.

(13) C. Bannwarth, E. Caldeweyher, S. Ehlert, A. Hansen, P. Pracht, J. Seibert, S. Spicher, S. Grimme *WIREs Comput. Mol. Sci.*, **2020**, 11, e01493. https://doi.org/https://doi.org/10.1002/wcms.1493.

(14) Neese, F. Software Update: The ORCA Program System, Version 4.0. *WIREs Comput. Mol. Sci.* **2018**, *8* (1), e1327. https://doi.org/https://doi.org/10.1002/wcms.1327.

(15) Becke, A. D. A New Mixing of Hartree–Fock and Local Density-functional Theories. *J. Chem. Phys.* **1993**, *98* (2), 1372–1377. https://doi.org/10.1063/1.464304.

(16) Weigend, F.; Ahlrichs, R. Balanced Basis Sets of Split Valence, Triple Zeta Valence and Quadruple Zeta Valence Quality for H to Rn: Design and Assessment of Accuracy. *Phys. Chem. Chem. Phys.* **2005**, *7* (18), 3297–3305. https://doi.org/10.1039/B508541A.

(17) Neese, F.; Wennmohs, F.; Hansen, A.; Becker, U. Efficient, Approximate and Parallel Hartree–Fock and Hybrid DFT Calculations. A 'Chain-of-Spheres' Algorithm for the Hartree–Fock Exchange. *Chem. Phys.* **2009**, *356* (1), 98–109. https://doi.org/https://doi.org/10.1016/j.chemphys.2008.10.036.

(18) Weigend, F. Accurate Coulomb-Fitting Basis Sets for H to Rn. *Phys. Chem. Chem. Phys.* **2006**, *8* (9), 1057–1065. https://doi.org/10.1039/B515623H.

(19) Pappas, D. K.; Borfecchia, E.; Dyballa, M.; Pankin, I. A.; Lomachenko, K. A.; Martini, A.; Signorile, M.; Teketel, S.; Arstad, B.; Berlier, G.; Lamberti, C.; Bordiga, S.; Olsbye, U.; Lillerud, K. P.; Svelle, S.; Beato, P. Methane to Methanol: Structure-Activity Relationships for Cu-CHA. *J. Am. Chem. Soc.* **2017**, *139* (42), 14961–14975. https://doi.org/10.1021/jacs.7b06472.

(20) Martini, A.; Borfecchia, E.; Lomachenko, K. A.; Pankin, I. A.; Negri, C.; Berlier, G.; Beato, P.; Falsig, H.; Bordiga, S.; Lamberti, C. Composition-Driven Cu-Speciation and Reducibility in Cu-CHA Zeolite Catalysts: A Multivariate XAS/FTIR Approach to Complexity. *Chem. Sci.* **2017**, *8* (10), 6836–6851. https://doi.org/10.1039/C7SC02266B.

(21) Buono, C.; Martini, A.; Pankin, I. A.; Pappas, D. K.; Negri, C.; Kvande, K.; Lomachenko, K. A.; Borfecchia, E. Local Structure of Cu(I) Ions in the MOR Zeolite: A DFT-Assisted XAS Study. *Radiat. Phys. Chem.* **2020**, *175*, 108111. https://doi.org/https://doi.org/10.1016/j.radphyschem.2018.12.031.

(22) Zabinsky, S. I.; Rehr, J. J.; Ankudinov, A.; Albers, R. C.; Eller, M. J. Multiple-Scattering Calculations of x-Ray-Absorption Spectra. *Phys. Rev. B* **1995**, *52* (4), 2995–3009. https://doi.org/10.1103/PhysRevB.52.2995.

(23) Martini, A.; Bugaev, A. L.; Guda, S. A.; Guda, A. A.; Priola, E.; Borfecchia, E.; Smolders, S.; Janssens, K.; De Vos, D.; Soldatov, A. V. Revisiting the Extended X-ray Absorption Fine Structure Fitting Procedure through a Machine Learning-Based Approach. *J. Phys. Chem. A* **2021**, *125* (32), 7080–7091. https://doi.org/10.1021/acs.jpca.1c03746.

(24) Fornasini, P. Introduction to X-Ray Absorption Spectroscopy, *in Synchrotron Radiation, Mobilio S., Boscherini F. and Meneghini C. Eds.,* **2015**, pp. 181-211. https://doi.org/10.1007/978-3-642-55315-8.

(25) Dutta, P. K.; Rao, K. M.; Park, J. Y. Correlation of Raman Spectra of Zeolites with Framework Architecture. *J. Phys. Chem.* **1991**, *95* (17), 6654–6656. https://doi.org/10.1021/j100170a050.

(26) Twu, J.; Dutta, P. K.; Kresge, C. T. Vibrational Spectroscopic Examination of the Formation of Mordenite Crystals. *J. Phys. Chem.* **1991**, *95* (13), 5267–5271. https://doi.org/10.1021/j100166a064.

(27) Signorile, M.; Bonino, F.; Damin, A.; Bordiga, S. UV-Raman Fingerprint of Brønsted Sies in MFI Zeolites: A Useful Marker in Dealumination Detection. *J. Phys. Chem. C* **2016**, *120* (32), 18088–18092. https://doi.org/10.1021/acs.jpcc.6b05520.

(28) Humbert, B.; Carteret, C.; Burneau, A.; Gallas, J. P. Fourier Transform Infrared and Raman Spectroscopic Study of Silica Surfaces. *In Colloidal Silica: Fundamentals and Applications;* Bergna, H.E., Roberts, W. O., Eds.; CRC Press, **2005**; pp 295–310.

(29) Vanelderen, P.; Snyder, B. E. R.; Tsai, M.-L.; Hadt, R. G.; Vancauwenbergh, J.; Coussens, O.; Schoonheydt, R. A.; Sels, B. F.; Solomon, E. I. Spectroscopic Definition of the Copper Active Sites in Mordenite: Selective Methane Oxidation. *J. Am. Chem. Soc.* **2015**, *137* (19), 6383–6392. https://doi.org/10.1021/jacs.5b02817.
